# Liver gene therapy by lentiviral vectors reverses anti-factor IX pre-existing immunity in haemophilic mice

**DOI:** 10.1002/emmm.201302857

**Published:** 2013-09-16

**Authors:** Andrea Annoni, Alessio Cantore, Patrizia Della Valle, Kevin Goudy, Mahzad Akbarpour, Fabio Russo, Sara Bartolaccini, Armando D'Angelo, Maria Grazia Roncarolo, Luigi Naldini

**Affiliations:** 1 TIGET, San Raffaele Telethon Institute for Gene Therapy, San Raffaele Scientific InstituteMilan, Italy; 2 Vita Salute San Raffaele UniversityMilan, Italy; 3 Coagulation Service and Thrombosis Research Unit, San Raffaele Scientific InstituteMilan, Italy

**Keywords:** gene therapy, haemophilia, immune tolerance

## Abstract

A major complication of factor replacement therapy for haemophilia is the development of anti-factor neutralizing antibodies (inhibitors). Here we show that liver gene therapy by lentiviral vectors (LVs) expressing factor IX (FIX) strongly reduces pre-existing anti-FIX antibodies and eradicates FIX inhibitors in haemophilia B mice. Concomitantly, plasma FIX levels and clotting activity rose to 50–100% of normal. The treatment was effective in 75% of treated mice. FIX-specific plasma cells (PCs) and memory B cells were reduced, likely because of memory B-cell depletion in response to constant exposure to high doses of FIX. Regulatory T cells displaying FIX-specific suppressive capacity were induced in gene therapy treated mice and controlled FIX-specific T helper cells. Gene therapy proved safer than a regimen mimicking immune tolerance induction (ITI) by repeated high-dose FIX protein administration, which induced severe anaphylactoid reactions in inhibitors-positive haemophilia B mice. Liver gene therapy can thus reverse pre-existing immunity, induce active tolerance to FIX and establish sustained FIX activity at therapeutic levels. These data position gene therapy as an attractive treatment option for inhibitors-positive haemophilic patients.

→See accompanying article http://dx.doi.org/10.1002/emmm.201302859

## INTRODUCTION

Haemophilia is a monogenic disease due to mutations in the gene encoding for coagulation factor VIII (FVIII; haemophilia A) or factor IX (FIX; haemophilia B). As a result, the deficiency or dysfunction of one of these factors impairs proper blood coagulation (Mannucci & Tuddenham, 2001). Haemophilic patients are currently treated by prophylactic or on-demand intravenous (i.v.) infusions of recombinant factors (replacement therapy) (Berntorp & Shapiro, 2012). The major complication of factor replacement therapy is the formation of antibodies (Abs) against the supplied factor that can neutralize its activity. Neutralizing anti-factor Abs are frequently referred to as inhibitors. Inhibitors develop in 20–30% of patients with severe haemophilia A and 3–5% of patients with haemophilia B following replacement therapy (Astermark et al, 2008).

Treatment of inhibitor-positive haemophilic patients is challenging since it must control bleeding episodes and eradicate the inhibitors. The most effective approach for eradicating inhibitors is immune tolerance induction (ITI). ITI is based, most often, on the daily administration of high doses of recombinant factor until the inhibitors disappear, which typically requires more than one year. Low-dose regimens have also been described. ITI has a success rate in the range of 60% for haemophilia A and 15–30% for haemophilia B (DiMichele, 2007). ITI is very expensive, demanding and entails the risk of developing anaphylaxis or nephrotic syndrome (Astermark et al, 2010; Ewenstein et al, 1997; Warrier et al, 1997). Because of the lower frequency of inhibitor development, there is less experience in the management of inhibitor in patients with haemophilia B. ITI can be attempted in these patients, but the risk of complications is higher than in haemophilia A (Benson et al, 2012; DiMichele et al, 2007). The mechanism by which ITI acts is not completely understood. It has been hypothesized that chronic exposure to the antigen (Ag) in ‘non-dangerous’ conditions (without concomitant activation of innate immunity) induces immune tolerance (Matzinger, 1994). Induction of anergy or apoptosis of memory B and T cells have been reported (Reipert et al, 2007). The management of patients who failed ITI is very challenging: classic immune suppression or administration of monoclonal anti-CD20 antibodies are generally ineffective (Fox et al, 2006; Mathias et al, 2004). Inhibitors increase both morbidity and mortality in haemophilia and represent a still unmet medical need.

Recently, gene therapy was shown to provide a promising treatment for haemophilia B, by establishing long-term expression of FIX in patients administered with a single i.v. dose of adeno-associated viral (AAV) vectors expressing functional FIX (High, 2012; Nathwani et al, 2011). Lentiviral vectors (LVs) are attractive tools for liver gene therapy, by virtue of their ability to stably integrate in the genome of target cells and the absence of pre-existing humoral and cellular immunity against vector components in most humans. We have previously reported long-term phenotypic correction of haemophilia B and induction of FIX-specific immune tolerance after a single i.v. administration of LVs to haemophilic mice, provided that transgene expression is stringently targeted to hepatocytes (Annoni et al, 2009; Brown et al, 2007; Cantore et al, 2012; Matrai et al, 2011). Targeting of transgene expression to hepatocytes is achieved by a combination of transcriptional control, mediated by a synthetic hepatocyte-specific promoter and post-transcriptional control obtained by adding to the transgene sequences complementary to the haematopoietic-specific microRNA 142, which binds and targets for degradation any vector mRNA ectopically expressed in antigen presenting cells of liver and spleen (Brown et al, 2006). In those study tolerance was achieved in mice naive to FIX. Here we set out to challenge this tolerogenic outcome by treating mice with induced pre-existing FIX inhibitors, in order to test the potential of liver gene therapy to reverse an established immune response. If successful, this approach may become a preferred therapeutic option for inhibitors-positive haemophilic patients.

## RESULTS

### FIX immunization induces anti-FIX Abs and inhibitors in haemophilia B mice

**Figure 1:**
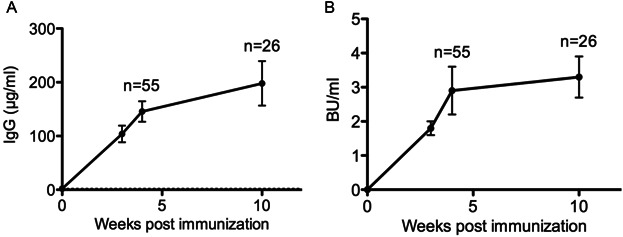
Humoral immune response after FIX immunization in haemophilia B mice Anti-FIX IgG Abs measured by ELISA in plasma samples collected from mice at the indicated times after FIX immunization. Data are presented as mean ± standard error of the mean (SEM).FIX inhibitors measured by Bethesda Assay in plasma samples collected from mice at the indicated times after FIX immunization. Data are presented as mean ± standard error of the mean (SEM).

In order to model inhibitors-positive patients, we induced an anti-FIX immune response in adult haemophilia B (*F9* knock out) mice (*n* = 55) in C57Bl/6 genetic background by subcutaneous administration of recombinant human FIX protein in incomplete Freund's adjuvant. All immunized mice mounted a robust humoral response resulting in an average 146 and 198 µg/ml of anti-FIX binding immunoglobulin G (IgG) at 4 and 10 weeks after immunization, respectively (Fig 1A). All mice developed FIX inhibitors at an average of 3 Bethesda units (BU)/ml with 15% of the mice reaching >5 BU/ml (Fig 1B and Supporting Information Fig S2D,H). The majority of anti-FIX IgG were of the IgG1 isotype, indicating a T helper type-2 (Th2)-skewed immune response (Supporting Information Fig S1A,B). We did not detect anti-FIX IgE in most of the mice. As expected from the natural course of a humoral immune response, we detected anti-FIX IgG secreting B cells mainly in the lymph-nodes (LNs) draining the immunization site at 4 weeks after immunization, whereas FIX-specific plasma cells (PCs) became detectable in the bone marrow (BM) at 10 weeks (Supporting Information Fig S1C,D).

### Liver gene therapy reverses anti-FIX Abs and eradicates inhibitors

**Figure 2:**
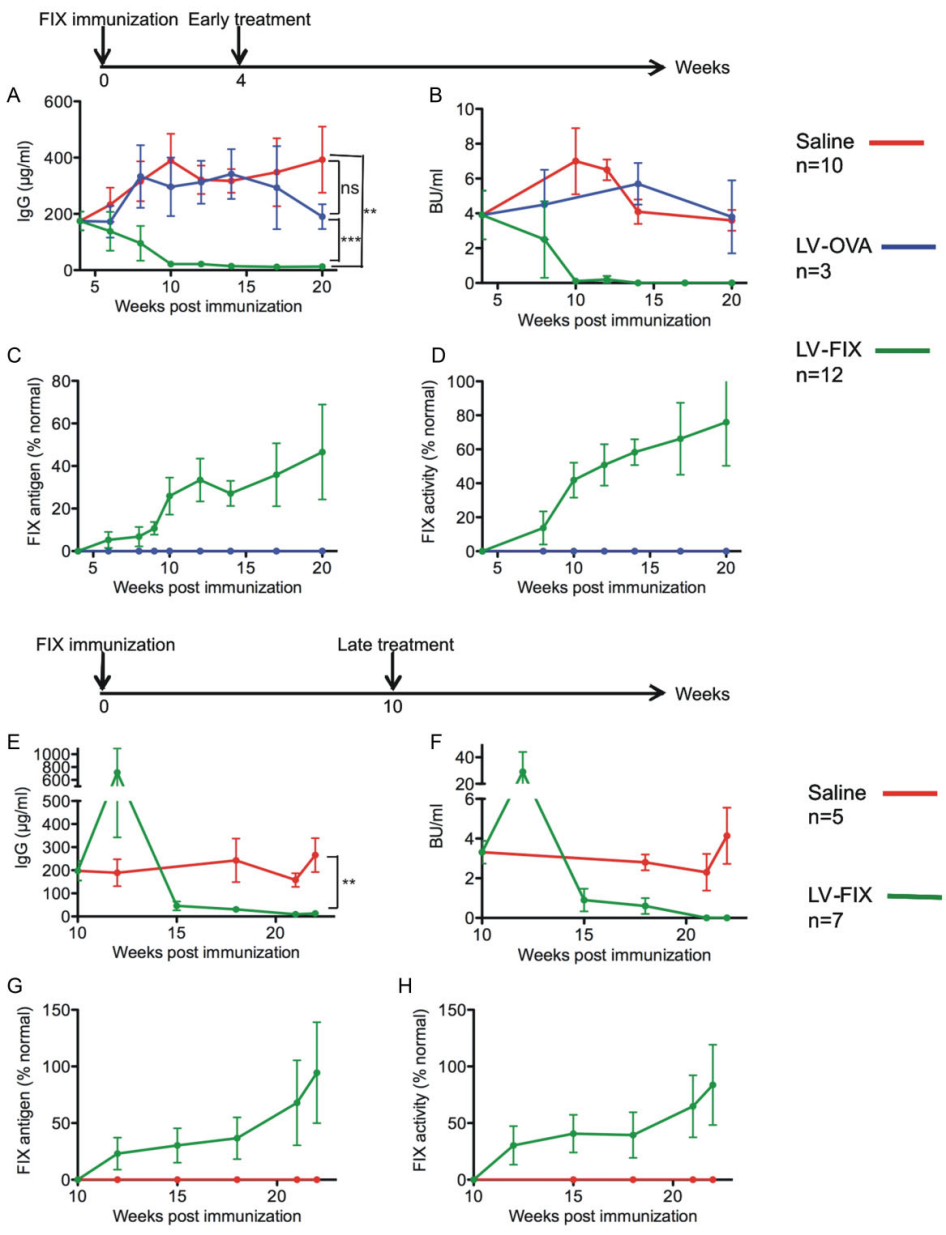
Anti-FIX humoral immune response and FIX expression after liver gene therapy in inhibitors-positive haemophilia B mice For the early treatment, mice were injected with saline (*n* = 10, red line) or 0.75–1 × 10^9^ TU/mouse of LV-FIX (*n* = 12 responders, green line) or LV-OVA (*n* = 3, blue line) 4 weeks after immunization (A–D). For the late treatment, mice were injected with saline (*n* = 5, red line) or 1–1.5 × 10^9^ TU/mouse of LV-FIX (*n* = 7 responders, green line) 10 weeks after FIX immunization (E–H). Data are presented as mean ± SEM. ns: not significant; **: *p* < 0.01; ***: *p* < 0.001 (*t* test). When data were scrutinized for each individual mouse, detectable FIX in the plasma correlated with lower level of anti-FIX binding antibodies (see also Supporting Information Fig 2). **A,E.** Anti-FIX IgG Abs measured by ELISA in plasma samples collected from mice at the indicated times after FIX immunization.**B,F.** FIX inhibitors measured by Bethesda Assay in plasma samples collected from mice at the indicated times after FIX immunization.**C,G.** FIX expression measured by ELISA in plasma samples collected from mice at the indicated times after FIX immunization.**D,H.** FIX activity measured by aPTT in plasma samples collected from mice at the indicated times after FIX immunization.

We then evaluated the impact of gene therapy on these inhibitors-positive haemophilia B mice. Four weeks after immunization (early treatment), mice were administered i.v. with LVs expressing either human FIX (*n* = 16 in 3 independent experiments) or the unrelated antigen chicken ovalbumin (OVA; *n* = 3), under the control of a synthetic hepatocyte-specific promoter (Enhanced Transthyretin, ET) and carrying microRNA-142 target sequences (Cantore et al, 2012) at doses of 0.75–1 × 10^9^ Transducing Units (TU)/mouse. A third group of mice was injected with vehicle only (saline, *n* = 10). In sharp contrast to saline- and LV-OVA injected mice, in which titers of anti-FIX Abs continued to increase and inhibitors remained high and stable (up to 7 BU/ml) throughout the follow up, 75% of the mice receiving LV-FIX showed a gradual decline in anti-FIX Abs over the 16 weeks following gene therapy, resulting in approximately 30-fold lower concentration than those observed in the other 2 groups (Fig 2A). In these mice, inhibitors became undetectable since 10 weeks after gene therapy (Fig 2B). Concomitantly with the decrease in anti-FIX Abs, FIX expression gradually rose, reaching more than 50% of normal levels at 16 weeks after gene therapy, which is in line with the expected reconstitution for the administered LV dose (Fig 2C,D). The degree of FIX reconstitution was similar when measured by antigen immunocapture or clotting activity of the mice plasma, further indicating that the residual anti-FIX Abs still detectable after gene therapy were not neutralizing. As mentioned above, gene therapy was not effective in all treated mice; 4 out of 16 mice, here defined as non-responders (NRs), failed to recover FIX expression and activity and 3 of them maintained FIX inhibitors in the plasma (Table 1). These NR mice showed a sharp increase, rather than decrease, in anti-FIX IgG concentration and FIX inhibitors in the first weeks after gene therapy (Supporting Information Fig S2A–D).

**Table 1:** Mice treated and responders to therapy

Treatment	Total number	BU/ml = 0	FIX activity >1% of normal	VCN
LV-FIX early treatment	16	13 (81%)	12 (75%)	1.5 ± 0.1
LV-FIX late treatment	10	7 (70%)	7 (70%)	3.2 ± 0.6
LV-OVA control	3	0 (0%)	0 (0%)	1.8 ± 0.5
Saline control	15	0	0	ND

The table shows the number of treated mice, the number (% of treated) of those having BU/ml = 0 and FIX activity > 1% of normal at the end of experiments (12–16 weeks after treatment or 20–22 weeks after immunization). Mice having BU/ml = 0 and FIX activity > 1% are defined responders to gene therapy and are shown in Fig 2. Mice having detectable BU/ml and/or FIX activity <1% of normal are defined non-responders (NRs) to gene therapy and are shown in Supporting Information Fig S2 as single mice. Vector copies per diploid genome (vector copy number, VCN) measured on DNA from the liver of treated mice are shown. ND: not detectable.

We then administered gene therapy to haemophilia B mice at a later time (10 weeks) after immunization (late treatment), when the anti-FIX humoral response was fully developed. Whereas saline-injected mice (*n* = 5) showed stable high concentration of anti-FIX IgG (>250 μg/ml) and inhibitors (4 BU/ml) throughout the follow-up time, LV-FIX treated mice (*n* = 10 in 2 independent experiments) showed an initial surge followed by a sharp decline of both anti-FIX IgG and inhibitors, which decreased to <10 μg/ml and to undetectable levels, respectively. As observed with the early treatment group, inhibitors disappeared in these mice and FIX expression and activity were reconstituted in the plasma to matching near-normal levels. Note that a slightly higher LV dose was administered in this case (1–1.5 × 10^9^ TU/mouse). The NR rate was similar to that observed after the early treatment (3 out of 10 did not recover FIX activity and maintained FIX inhibitors (Table 1 and Supporting Information Fig S2E–H). Intriguingly, NR mice had significantly lower vector content in the liver than responder mice at the end of the experiments.

We also found a consistent decrease in anti-FIX IgG1, IgG2a and IgG2b in LV-FIX treated responders mice as compared to saline-injected controls both after early and late treatment, further supporting effective reversal of anti-FIX humoral immunity (Supporting Information Fig S3).

We analysed the kinetics of appearance of circulating FIX after gene therapy in naive or pre-immunized haemophilia B mice as well as the kinetics of disappearance of anti-FIX IgG. While FIX expression reaches its plateau levels 4 weeks after gene therapy in naive haemophilia B mice (Brown et al, 2007; Cantore et al, 2012), FIX expression raises more slowly in pre-immunized haemophilia B mice after gene therapy, with an initial delay during which anti-FIX Abs concentration drops to residual levels. Only after this drop, FIX expression gradually raises with a slower kinetics in mice treated at a later time after immunization (Supporting Information Fig S4).

### Repeated high-dose FIX protein injections induce anaphylactoid reactions in inhibitors-positive haemophilia B mice

**Figure 3:**
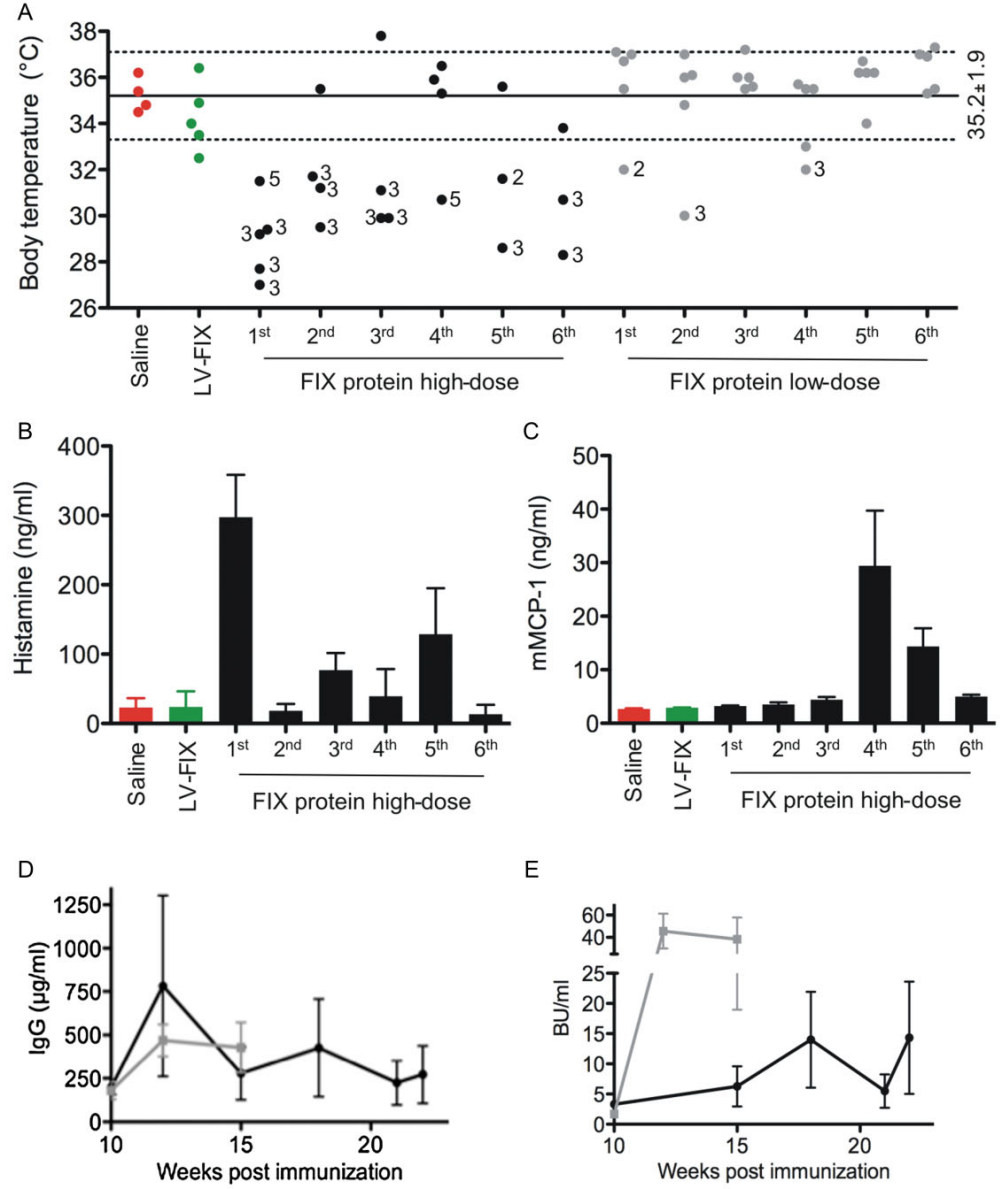
Anaphylactoid reactions and anti-FIX humoral immune response after repeated FIX protein administrations in inhibitors-positive haemophilia B mice Mice were treated with i.v. recombinant FIX protein injections at high dose (200 IU/kg) or low dose (20 IU/kg) every other day for 2 weeks, starting at 10 weeks after FIX immunization (for a total of 6 administrations). **A.** Body temperature measured 30–45 minutes after saline (red circles, *n* = 5), LV-FIX (green circles, *n* = 5) or after each recombinant high-dose (black circles, *n* = 5) or low-dose (grey circles, *n* = 5) FIX protein administration. Data from single mice are plotted. The mean and SD (35.2 ± 1.9) of repeated measurement of body temperature in 6 naïve haemophilia B mice is shown. The allergy score is reported for each mouse when not 0. Allergy score was performed according to (Li et al, 2003): 0 = no symptoms; 1 = scratching and rubbing around the snout and head; 2 = puffiness around the eyes and snout, diarrhea, pilarerecti, reduced activity, and/or decreased activity with increased respiratory rate; 3 = wheezing, labored respiration, cyanosis around the mouth and the tail; 4 = no activity after prodding, or tremor and convulsion; 5 = death.**B,C.** Histamine and mouse mast-cell protease 1 (mMCP-1) measured by ELISA in plasma samples collected 30–45 min after saline (red bar, *n* = 5), LV-FIX (green bar, *n* = 5) or after each recombinant high-dose (black bars, *n* = 5) FIX protein administration. Data are presented as mean ± SEM.**D.** Anti-FIX IgG Abs measured by ELISA in plasma samples collected from mice treated with i.v. recombinant FIX protein injections at high dose (black line, *n* = 9) or low dose (grey line, *n* = 5) at the indicated times after FIX immunization. Data are presented as mean ± SEM.**E.** FIX inhibitors measured by Bethesda Assay in plasma samples collected from mice treated with i.v. recombinant FIX protein injections at high dose (black line, *n* = 9) or low dose (grey line, *n* = 5) at the indicated times after FIX immunization. Data are presented as mean ± SEM.

In order to compare the efficacy and safety of gene therapy *versus* ITI in reverting the anti-FIX antibody response, we administered haemophilia B mice an ITI-like regimen (consisting of high-dose recombinant FIX protein at 200 IU/kg i.v. every other day) starting at 10 weeks after immunization (*n* = 9 in 2 independent experiments). Four of these mice (45%) died during the course of the treatment. In some treated mice we monitored body temperature before and 30–45 min after vector or protein injection(s) and assigned an allergy score (Li et al, 2003; Sun et al, 2010). While gene therapy-treated mice did not show significant alteration of body temperature 30 min after LV injection and at every other day check point throughout the following 2 weeks, every high-dose FIX protein injection caused severe hypothermia, signs of anaphylactoid reactions, distress and in some cases death (Fig 3A). We evaluated whether these adverse events also occur following a lower-dose ITI like regimen. We treated haemophilia B mice by repeated i.v. administrations of recombinant FIX at 20 IU/kg, starting 10 weeks after immunization (*n* = 5). Even a 10-fold lower FIX dose induced hypothermia and signs of anaphylactoid reactions, although to a lesser extent than observed with higher doses (Fig 3A). We also observed an increase in plasma levels of histamine and mouse mast-cell protease 1 (mMCP-1) 30–45 min after most FIX injections as compared to saline and LV-FIX treatment (Fig 3B,C), suggesting a role of mast-cells in these IgE-independent anaphylactoid reactions (Finkelman et al, 2005). Because of the high morbidity and mortality of the high-dose regimen, we halted administrations of FIX protein 2 weeks after initiation. We measured anti-FIX IgG and inhibitor titers over the following 5–10 weeks in the surviving mice and did not find any difference in anti-FIX IgG, whereas inhibitor titers even increased (up to 40 BU/ml) as compared to levels measured before initiation of the treatment (Fig 3D,E). In order to determine the levels of circulating FIX achieved after a high- and low-dose of FIX protein administered for ITI-like regimens, we i.v. administered 200 or 20 IU/kg of recombinant FIX in naive haemophilia B mice (*n* = 4 *per* dose) and detected 46 and 2% of normal FIX levels, respectively, 1 h after administration. These levels declined by half in the next 6 h and became almost undetectable after 2 days (Supporting Information Fig S5). As expected, in inhibitors-positive haemophilia B mice we could not detect circulating FIX, when assessed at 1 h after protein administration.

### Anti-FIX Abs producer cells are reduced after liver gene therapy in inhibitors-positive haemophilia B mice

In order to investigate the mechanism responsible for the reduction of the anti-FIX Abs after liver gene therapy, we evaluated the B-cell response at the end of the experiments, approximately 20 weeks after immunization, when the reduction of anti-FIX Abs titer in gene therapy-treated mice was stable for several weeks (see Fig 2A,E).

**Figure 4:**
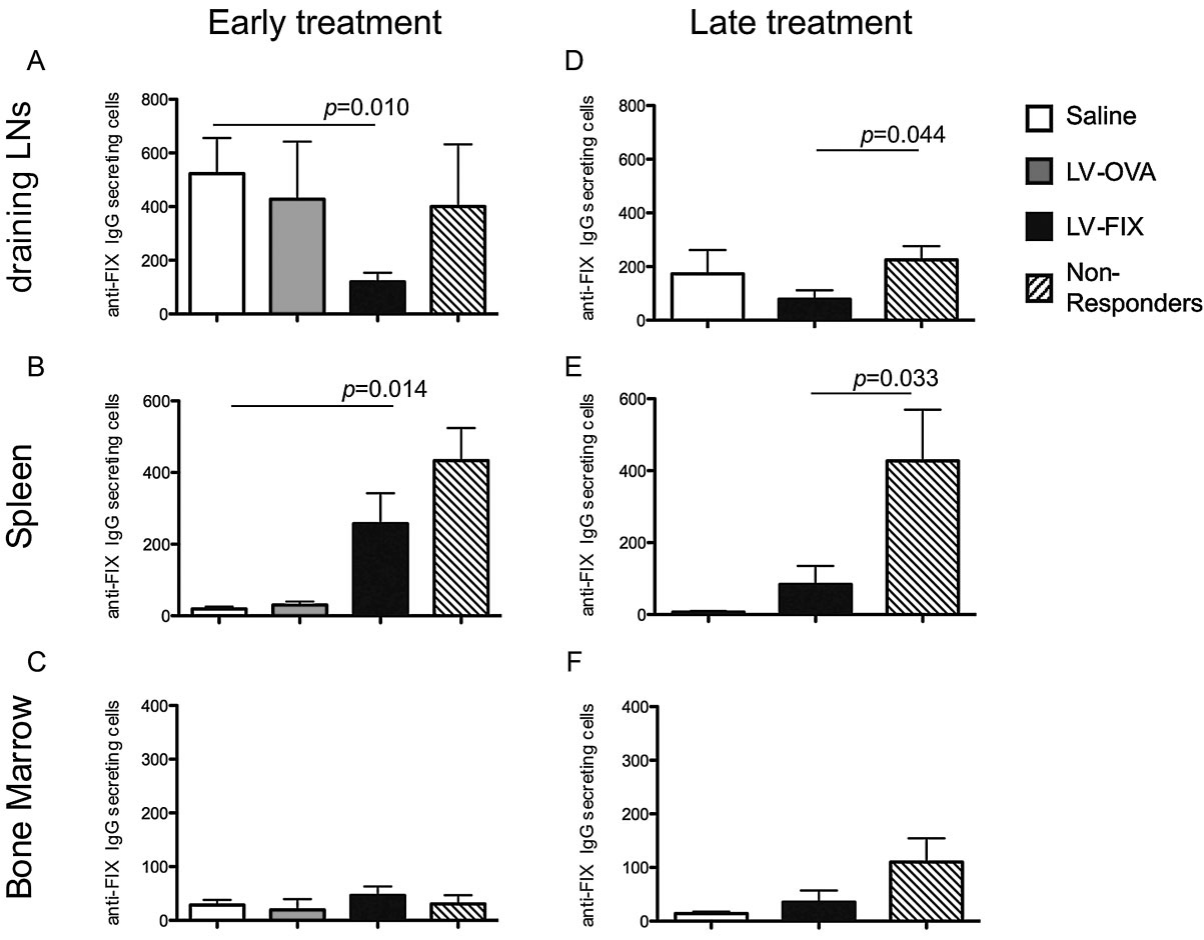
FIX-specific plasma cells after liver gene therapy in inhibitors-positive haemophilia B mice Data from 2 independent experiments for each experimental protocol are presented as mean number FIX-specific PC ± SEM for each experimental group, separating from LV-FIX treated mice those defined and presented as non-responders (NR, detectable BU/ml and/or FIX activity <1% of normal, see Table 1). **A–C.** FIX-specific IgG secreting plasma cells (PCs) out of 10^6^ total B or bone marrow cells were enumerated by elispot assay in the draining LNs, Spleen and Bone Marrow (as indicated) isolated from in inhibitors-positive haemophilia B mice 16 weeks after early treatment.**D–F.** FIX-specific IgG secreting plasma cells (PCs) out of 10^6^ total B or bone marrow cells were enumerated by elispot assay in the draining LNs, Spleen and Bone Marrow (as indicated) isolated from in inhibitors-positive haemophilia B mice 12 weeks after late treatment.

In agreement with plasma analyses, LV-FIX treatment (both early and late) led to a consistent reduction in the number of FIX-specific PCs in LNs draining the immunization site in responder mice. NR mice, on the contrary, displayed a number of anti-FIX IgG secreting PCs comparable to saline- and LV-OVA treated controls (Fig 4A,D). Intriguingly, the frequency of FIX-specific PCs increased in the spleen of gene therapy-treated mice compared to saline- and LV-OVA treated controls (Fig 4B,E), although this increase did not affect the efficacy of gene therapy in reducing circulating anti-FIX Abs. This data suggest that splenic FIX-specific PCs are responsible for the residual titer of anti-FIX Abs still detectable in these mice, despite the absence of FIX inhibition, and may have been induced by systemic exposure to the gene-delivered FIX Ag. Indeed, the increase in splenic FIX-specific PCs was even higher in mice NR to gene therapy. The frequency of PCs in the BM was not altered by any treatment, except for a slight increase in NR mice (Fig 4F).

Overall, these results indicate that eradication of anti-FIX neutralizing Abs is associated with inhibited generation of FIX-specific PCs in the LNs draining the site where the immune response to FIX was induced and the Ag persists. Successful eradication of FIX inhibitors by LV-FIX treatment did not completely clear anti-FIX Abs secreting cells, suggesting persistence of cells secreting non-inhibitory Abs. It is possible that the residual titers of binding anti-FIX Abs detected by immunocapture were not high enough to reach the threshold required to inhibit clotting enough to register on the Bethesda assay.

### Constant exposure to high FIX levels reduces memory B cells and prevents renewal of anti-FIX inhibitory Abs secreting cells

**Figure 5:**
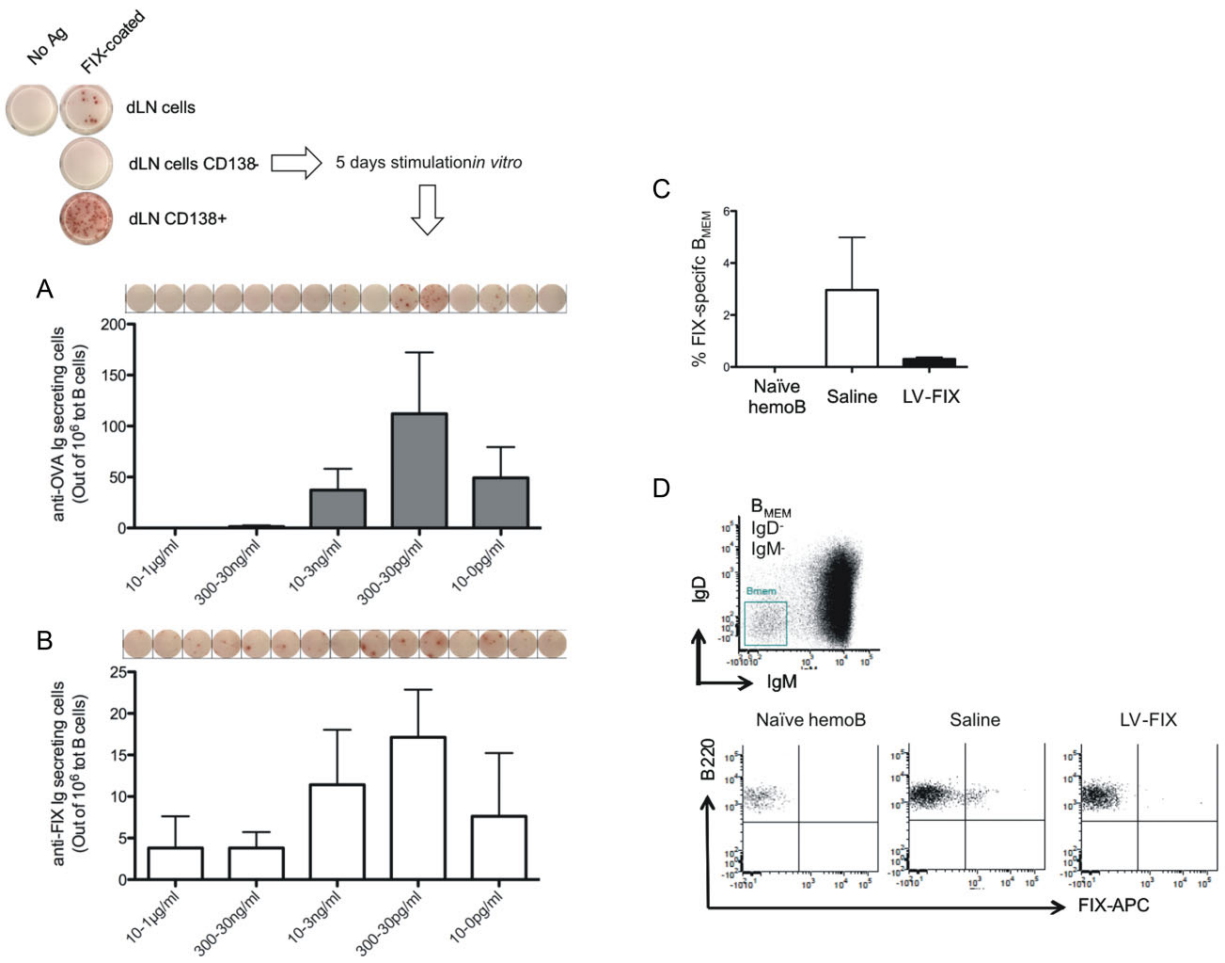
Activation and survival of pre-existing FIX specific B_MEM_ in response to different FIX concentrations Ten weeks after FIX or OVA immunization, LN cells were isolated and CD138^+^ PCs were removed. CD138^−^ LN cells were then cultured in the presence of the relevant Ag at doses ranging from 10µg/ml to 3pg/ml for 5 days in order to activate quiescent Ag-specific B_MEM_. **A.** OVA-specific IgG-secreting PCs, which derive from activated B_MEM_, were counted by elispotat the end of the Ag exposure. Data are presented as mean ± SEM.**B.** FIX-specific IgG-secreting PCs, which derive from activated B_MEM_, were counted by elispotat the end of the Ag exposure. Data are presented as mean ± SEM.**C–D.** The frequency of FIX-specific B_MEM_ (CD19^+^B220^+^IgD^−^IgM^−^FIX^+^_APC_) was evaluated by FACS in LN cells harvested at 12 weeks after late treatment (*n* = 1 naïve haemoB as control; *n* = 3 Saline-treated; *n* = 4 LV-FIX treated). Data are presented as mean ± SEM. A representative dot plot for each group is shown.

The activation of memory B cells (B_MEM_) at a second encounter with the Ag depends on the concentration of the cognate Ag and T helper (Th) cells. It has been shown that the persistence of high Ag levels leads to deletion of Ag-specific B_MEM_ (Hausl et al, 2005) and protein-based ITI protocols for inhibitors-positive haemophilic patients are designed on this rationale. In the mice treated in this study we reached FIX levels ranging from 2.5 to 5 µg/ml (50–100% of normal; see Fig 2). Therefore, we investigated whether these FIX levels may affect the function and survival of FIX-specific B_MEM_. To this aim, PC-depleted (CD138^-^) LN cells were isolated from mice immunized with OVA or FIX. These cells, which comprise quiescent Ag-specific B_MEM_, were restimulated *in vitro* for 5 days with increasing doses of the relevant Ag in order to define the range of Ag concentration leading to B_MEM_ activation (Fig 5A,B). Maximal activation of OVA- and FIX-specific B_MEM_ was observed at 30–300 ng/ml of Ag, whereas the number of Ag-specific activated B_MEM_ decreased at concentrations >300 ng and up to 10 µg/ml. To evaluate whether FIX generated *in vivo* by LV-FIX gene therapy inhibits the activation or even deplete FIX-specific B_MEM_, we measured the frequency of FIX-specific B_MEM_ (CD19^+^B220^+^IgM^−^IgD^−^) in LNs harvested from inhibitors-positive haemophilia B mice after LV-FIX gene therapy (Fig 5C,D). FIX-specific B_MEM_ were present in saline-injected mice, whereas they were barely detectable in gene therapy-treated mice. These results indicate that the *in vivo* reconstituted FIX after liver gene therapy depletes FIX-specific B_MEM_, leading to progressive contraction of the pool of PCs secreting high-affinity inhibitory anti-FIX IgG.

### The anti-FIX T-cell response is suppressed by Ag-specific Tregs

**Figure 6:**
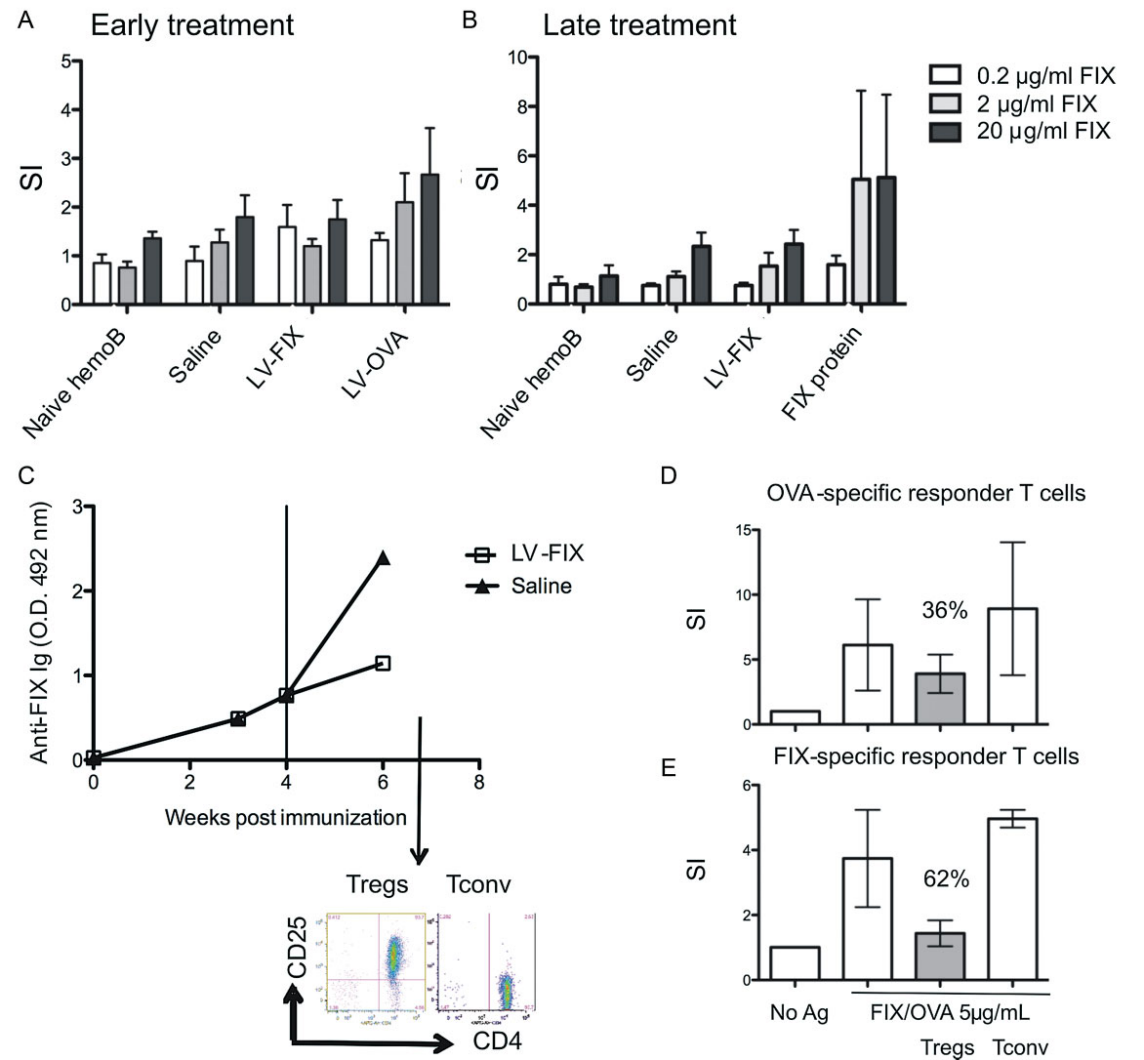
FIX-specific T-cell response and Tregs Ag-specific suppressive activity after LV-FIX gene therapy FIX-driven T-cell-proliferative response was measured stimulating B cells-depleted (B220^−^) splenocytes isolated from inhibitors-positive haemophilia B mice, 16 weeks after the early treatment.FIX-driven T-cell-proliferative response was measured stimulating B cells-depleted (B220^−^) splenocytes isolated from inhibitors-positive haemophilia B mice, 12 weeks after the late treatment.Proliferation data are presented as mean of Stimulation Index (SI) ± SEM for each experimental group (2 independent experiments for each treatment protocol), excluding from LV-FIX treated mice, those defined as non-responders (NR, detectable BU/ml and/or FIX activity <1% of normal).Regulatory T cells (CD4^+^CD25^+^ Tregs) and conventional T cells (CD4^+^CD25^−^Tconv) were isolated from inhibitors-positive haemophilia B mice 2–3 weeks after LV-FIX gene therapy (*n* = 3), at a time in which the anti-FIX Abs were lower than saline-injected controls (arrow). LV-FIX or saline were administered 4 weeks after immunization (line).LN cells derived from OVA-immunized mice were stimulated with the relevant Ag alone or in co-culture with Tregs and Tconv (ratio 1:0.5) to define their suppressive capacity. Data are presented as mean SI ± range for each culture condition (2 independent experiments).LN cells derived from FIX-immunized mice were stimulated with the relevant Ag alone or in co-culture with Tregs and Tconv (ratio 1:0.5) to define their suppressive capacity. Data are presented as mean SI ± range for each culture condition (2 independent experiments).

Th cells are crucial for the development of B-cell mediated responses and for activation of B_MEM_ (Shlomchik & Weisel, 2012). We evaluated whether the T-cell-mediated response to FIX was altered in inhibitors-positive mice in response to gene therapy (Fig 6A,B). FIX-specific T-cell-proliferative response was weak (∼2 index of stimulation, SI) but still detectable in mice at the end of the experiments, approximately 20 weeks after immunization. Neither the early or late LV-FIX gene therapy affected this response, as compared to saline- or LV-OVA treated controls. In agreement with the failure to reduce the inhibitory response to FIX, repeated high-dose protein administrations led to a more robust T-cell responsiveness. Although protein treatment was interrupted after 6 administrations, our data suggest that FIX protein-based treatment for tolerance induction in a primed immune system may boost the T-cell response, instead of suppressing it.

We have previously shown that LV-FIX gene therapy can establish a robust state of transgene-specific tolerance mediated by Ag-specific FoxP3^+^ Tregs (Annoni et al, 2009; Brown et al, 2007). Thus, we investigated whether FoxP3^+^ Tregs isolated from inhibitors-positive haemophilia B mice after gene therapy acquired *in vivo* FIX-specific suppressive capacity. To this aim, we isolated Tregs (CD4^+^CD25^+^) and effector T cells (CD4^+^CD25^−^) 3 weeks after administration of LV-FIX to pre-immunized haemophilia B mice (*n* = 3), when anti-FIX Abs were already reduced compared to saline-injected mice (Fig 6C). These cells were tested *in vitro* for suppression of Ag-driven proliferation of T cells harvested from immunized mice (Fig 6D,E). Tregs isolated from LV-FIX treated mice suppressed the proliferation of FIX-specific effector T cells while showing only modest activity on OVA-specific effector T cells stimulated by the cognate Ag.

Interleukin-10 (IL-10) producing B cells (B10) have been shown to regulate inflammation and autoimmune diseases as well as innate and antigen-specific adaptive immune responses (Fillatreau et al, 2002; Yoshizaki et al, 2012). We evaluated whether B10 are expanded in inhibitors-positive haemophilia B mice after gene therapy. We found no difference in the percentage of B10 in mice after LV-FIX treatment (*n* = 3) as compared to saline-injected controls (*n* = 2) and naive haemophilia B mice (*n* = 2), both upon a polyclonal (lipopolysaccharide, LPS), or FIX-specific stimulation (anti-CD40 and plate-bound FIX) (Supporting Information Fig S6). These data suggest that B10 are unlikely to be responsible for the regulation of the anti-FIX humoral response observed after gene therapy.

Overall, these results indicate that FIX-specific Th cells persist *in vivo* after eradication of FIX inhibitors and are responsive to the Ag *in vitro* in a range of concentration of 2–20 µg/ml, which overlaps with that provided *in vivo* after liver gene therapy. Importantly, these effector T cells are kept under control by FIX-specific FoxP3^+^ Tregs generated *in vivo* by LV-FIX gene therapy.

## DISCUSSION

A major complication of FIX replacement therapy is the development of inhibitors to the infused concentrate. Here, we show that LV-mediated FIX gene transfer to hepatocytes can reverse pre-existing neutralizing immunity in haemophilia B mice. Once the anti-FIX antibody response has declined, liver gene therapy provides sustained expression of FIX to therapeutic levels.

Our FIX immunization protocol generated robust anti-FIX immunity in all treated mice, which showed high titers of FIX-specific IgG1 and inhibitors in the plasma. FIX-specific B-cell response was mainly localized to the LNs draining the immunization site and was still detectable 22 weeks after immunization, suggesting persistent Ag stimulation and generation of Ag-specific PCs. Remarkably, a single administration of LV-FIX targeted for expression to hepatocytes strongly reduced anti-FIX Abs and eradicated inhibitors in 75% of the treated mice, when performed both at the time of development or during ongoing anti-FIX immune response.

The molar excess (30–60 fold) of anti-FIX IgG produced in the mice as compared to the FIX output observed after Abs reversal makes it unlikely that FIX expression *per se* can saturate circulating Abs and overcome their effect. This is true even in the case that Ag binding decreases the half-life of circulating Abs by triggering phagocytosis or trapping and deposition in basal membranes. Indeed, at the time when gene-delivered FIX has reached its maximal expression in naive mice, FIX expression in immunized mice is only at a small fraction of the final levels, suggesting substantial squelching by the circulating Abs (see Supporting Information Fig S4). This squelching gradually disappears after anti-FIX Abs reached the lower range of concentration, indicating halted supply and progressive depletion of circulating Abs. These findings indicate that gene therapy shuts down the humoral immune response at the cellular levels, *i.e*. targeting anti-FIX secreting PCs and B_MEM_.

Indeed, gene therapy-treated mice showed fewer anti-FIX Abs secreting PCs than controls in the LNs draining the immunization site. In these mice, we also found almost complete disappearance of FIX-specific B_MEM_. This finding suggests that gene therapy induced contraction of pre-existing FIX-specific B_MEM_, which, in turn, decreases anti-FIX Abs producing PCs and, as a consequence, the supply of circulating anti-FIX Abs. It also suggests that this effect was dependent on the dose of Ag and Ag-specific. A similar mechanism was invoked to explain eradication of inhibitors by ITI. It has been reported that sustained exposure to high levels FVIII induces apoptosis of FVIII-specific B_MEM_ in haemophilia A mice in which anti-FVIII Abs were induced (Hausl et al, 2005). Here we show that escalating FIX concentrations inhibit B_MEM_ reactivation *in vitro*. Once B_MEM_ have been shut down, the antibody response gradually declines, as PCs turnover.

In addition to inducing high levels of transgene-derived Ag, liver-directed gene therapy has the accompanying benefit of inducing transgene-specific Tregs. Others and we have already shown that hepatocyte-targeted expression of an Ag by different types of vectors favours the induction of tolerance mediated by Ag-specific Foxp3^+^ Tregs (Annoni et al, 2009; Cao et al, 2007; Matrai et al, 2011; Mingozzi et al, 2003). We now provide evidence that FIX-specific tolerance induction can be achieved in the setting of an already Ag-primed immune system. As Tregs can keep effector T cells under control, they likely inhibit FIX-specific Th in the treated mice. If gene therapy is administered during the development phase of the humoral response (early treatment), FIX-specific Tregs can interfere with B-cell maturation. If gene therapy is administered after the complete maturation of the FIX-specific humoral response (late treatment), these Tregs can block Th functions required for the re-activation of B_MEM_ after re-encounter with the Ag. We noticed that systemic exposure to FIX induced by gene therapy resulted in an increase in FIX-specific PCs in the spleen, which may be responsible for the residual level of FIX Abs found in responder mice. Since these cells developed after gene therapy, during the concomitant induction of FIX-specific tolerance, it is possible that Tregs interfered with their affinity maturation, thus explaining the absence of neutralizing anti-FIX Abs, which inhibit FIX activity.

It has been also shown that Tregs may directly control B-cell responses *in vivo* and *in vitro*, by a cell-contact dependent mechanism, which leads to B cells death and involves the release of cytotoxic molecules (Iikuni et al, 2009). Further investigations will be required to elucidate whether this mechanism is also involved in the inhibition of the anti-FIX B-cell response. As LV-OVA treatment did not affect the anti-FIX humoral response, despite being able to induce OVA-specific Tregs (Matrai et al, 2011), we conclude that Tregs mode of action is mainly Ag-specific, also in the case of a direct regulation of anti-FIX B-cell response. Whether a similar Ag-specific active tolerance mediated by Tregs occurs also after ITI remains to be defined. Overall, our results support the idea that hepatocyte-targeted Ag expression represents a robust approach to establish Ag-specific tolerance and suppress an ongoing immune reaction *in vivo*.

A fraction of inhibitors-positive mice failed to respond to gene therapy. We did not find any obvious difference in inhibitors titer at the time of treatment between NR and responders mice and we have evidence that inhibitors up to 32 BU/ml can be eradicated in our settings (see Supporting Information Fig S2D). It is possible that NR mice did not achieve the threshold of FIX expression required to halt the immune response, due to variability in transduction efficiency. This hypothesis is supported by the finding of an average lower vector content in the liver of NR *versus* responder mice. In NR mice we found a higher frequency of anti-FIX Abs secreting PCs than in saline-injected controls, suggesting that re-challenge with Ag at levels lower than a certain threshold can expand the FIX-specific B cells compartment. This is also in line with what was observed in responder mice treated with LV-FIX gene therapy at late times after immunization. In these mice, anti-FIX IgG started to decline only after an initial up rise. This observation may reflect an initial expansion of FIX-specific B_MEM_ induced by low Ag concentration, followed by B_MEM_ depletion due to increasing concentration of Ag, which leads to the contraction of the immune response. Further studies exploring the dose-dependent response of gene therapy for inhibitor reversal will address this point.

We showed that haemophilia B mice with anti-FIX Abs can experience severe anaphylactoid reactions upon i.v. injections of recombinant FIX protein. This finding has been previously reported for FIX as well as other therapeutic proteins injected in mice with pre-existing specific Abs (Sun et al, 2010; Verma et al, 2010). On the contrary, gene-delivered FIX did not induce such adverse effects, possibly due to its sustained expression rather than spiking pattern of FIX appearance following i.v. administration. Liver gene therapy may therefore be more effective and safer than ITI in eradicating FIX inhibitors and induce tolerance, at least in mouse models. In addition, gene therapy establishes sustained endogenous expression of the clotting factor and thus corrects the disease. It has been recently reported that liver gene therapy with AAV vectors can establish canine FVIII expression in haemophilia A dogs with pre-existing anti-FVIII Abs (Finn et al, 2010; Scott & Lozier, 2012). This therapy also resulted in a gradual decline of anti-FVIII Abs titer in treated dogs over the course of 1 year. In an independent study reported by Markusic et al, AAV8-derived vector was used to eradicate FIX inhibitors in haemophilia B mice (Markusic et al, 2013). Importantly, similar results were obtained by Markusic et al and our group using different vector platforms and mice with different genetic background indicating that liver-directed gene therapy is a robust strategy to reverse pre-existing immunity in haemophilia B. Both papers show that FIX-specific memory B cells are less prone to re-activate upon exposure to FIX concentration higher than 300 ng/ml (corresponding to 6% of normal levels). An apparent discrepancy between the two studies is that, while Markusic et al reported reversal of FIX inhibitors by AAV-mediated gene therapy at only 6% of normal FIX levels, we report the same outcome by LV-mediated gene therapy at 50–100% of normal FIX levels. Markusic et al used FIX knock-out mice with a different genetic background and i.v. injections of FIX for immunization instead of subcutaneous administration in incomplete Freund's adjuvant. Likely for these reasons, anti-FIX IgG concentrations are lower in the Markusic study, reaching approximately 30 µg/ml at the time of gene therapy administration, while in our work they reach approximately 200 µg/ml (see Fig 2A and E). As we postulate that reversal of the B-cell response is achieved only above a certain threshold of circulating FIX concentration, it is possible that the initial bioavailability of FIX is lower in our model, due to the higher concentration of binding anti-FIX Abs. Thus, pre-treatment regimens aiming to transiently deplete circulating Abs and/or B cells before gene therapy may also increase the success rate and, possibly, decrease the required vector dose, by favouring early antigen bioavailability. In addition, such treatments should alleviate the risks of inducing immune complex formation and related pathology.

In conclusion, our study candidates LV-mediated liver gene therapy as an attractive therapeutic strategy for inhibitors-positive haemophilic patients, for which the current treatments are unsatisfactory.

## MATERIALS AND METHODS

### Vector construction and production

Plasmid construction was carried out using standard cloning techniques. The plasmids pCCLsin.cPPT.ET.FIX.wpre.142T and pCCLsin.cPPT.ET.OVA.wpre.142T were previously described (Brown et al, 2007; Matrai et al, 2011). Third-generation LVs were produced by calcium phosphate transient transfection of 293T cells of the selected transfer vector, the packaging plasmid pMDLg/p.RRE, pCMV.REV, the VSV-G envelope plasmid pMD2.G and the pAdVAntage plasmid (Promega), as previously described (Follenzi & Naldini, 2002). 293T cells are seeded 24 h before transfection in 15-cm dishes. Two hours before transfection culture medium is replaced with fresh medium. For each dish, a solution containing a mix of the selected transfer plasmid, the packaging plasmids pMDLg/pRRE and pCMV.REV, pMD2.G and the pAdVAntage plasmid is prepared using 35, 12.5, 6.25, 9 and 15 µg of plasmid DNA, respectively. A 0.1× TE solution (10 mM Tris-HCl, 1 mM EDTA pH 8.0 in dH_2_O) and water (1:2) is added to the DNA mix to 1250 µl of final volume. The solution is left on a spinning wheel for 20–30 min, then 125 µl of 2.5 M CaCl_2_ are added. Right before transfection, a precipitate is formed by adding 1250 µl of 2× HBS (281 mM NaCl, 100 mM HEPES, 1.5 mM Na_2_HPO_4_, pH 7.12) while the solution is kept in agitation on a vortex. The precipitate is immediately added to the culture medium and left on cells for 14–16 h and after that the culture medium is changed. Supernatant is collected 30 h after medium change and passed through a 0.22 µm filter (Millipore). Filtered supernatant is transferred into sterile 25 mm × 89 mm poliallomer tubes (Beckman) and centrifuged at 20,000*g* for 120 min at 20°C (Beckman Optima XL-100K Ultracentrifuge). Vector pellet is dissolved in the appropriate volume of saline solution to allow a 500× concentration.

### Vector titration

293T cells were transduced with serial vector dilutions in the presence of polybrene (16 µg/ml). Genomic DNA (gDNA) was extracted 14 days after transduction, by using Maxwell 16 Cell DNA Purification Kit (Promega), according to manufacturer's instructions. Vector copies per diploid genome (vector copy number, VCN) were quantified by quantitative PCR (qPCR) starting from 100 ng of template gDNA using primers (HIV sense: 5′-TACTGACGCTCTCGCACC-3′; HIV antisense: 5′-TCTCGACGCAGGACTCG-3′) and a probe (FAM 5′-ATCTCTCTCCTTCTAGCCTC-3′) against the primer binding site region of LVs. Endogenous DNA amount was quantified by a primers/probe set against the human telomerase gene (Telo sense: 5′-GGCACACGTGGCTTTTCG-3′; Telo antisense: 5′-GGTGAACCTCGTAAGTTTATGCAA-3′; Telo probe: VIC 5′-TCAGGACGTCGAGTGGACACGGTG-3′ TAMRA). Copies per genome were calculated by the formula = [ng LV/ng endogenous DNA] × [no of LV integrations in the standard curve]. The standard curve was generated, by using a CEM cell line stably carrying four vector integrants, which were previously determined by Southern blot and FISH analysis. All reactions were carried out in duplicate or triplicate in an ABI Prism 7900HT Realtime PCR thermal cycler (Applied Biosystems). Each qPCR run carries an internal control generated by using a CEM cell line stably carrying 1 vector integrant, which were previously determined by Southern blot and FISH analysis. Titer is expressed as TU_293T_ (TU)/ml and calculated using the formula TU/ml = [VCN × 10^5^ × 1/dilution factor].

### Mice experiments and allergy score

Founder C57BL/6 *F9* knock out (haemophilia B) mice were obtained from the laboratory of Dr. Inder Verma at the Salk Institute (Wang et al, 1997). All the mice were maintained in specific-pathogen free conditions. FIX immunization was carried out by subcutaneous administration of 40 µg of recombinant human FIX protein (Pfizer) in incomplete Freund adjuvant (Sigma), in adult (7–10 weeks old) mice. LV and recombinant human FIX protein administration was carried out by tail-vein injections. Mice were bled from the retro-orbital plexus using capillary tubes and blood was collected into 0.38% sodium citrate buffer, pH 7.4. Body temperature was measured by a rectal probe and allergy score was assigned 30–45 min after LV or protein administration according to the following parameters: 0 = no symptoms; 1 = scratching and rubbing around the snout and head; 2 = puffiness around the eyes and snout, diarrhoea, pillar erecti, reduced activity and/or decreased activity with increased respiratory rate; 3 = wheezing, laboured respiration, cyanosis around the mouth and the tail; 4 = no activity after prodding, or tremor and convulsion; 5 = death (Li et al, 2003). Mice were anaesthetized with tribromoethanol (Avertin) and euthanized by CO_2_ inhalation at the expected time points. All animal procedures were performed according to protocols approved by the Institutional Animal Care and Use Committee.

### Enzyme-linked immunosorbent assays (ELISA) and coagulation assays

The concentration of human FIX was determined in mouse plasma by ELISA specific for human FIX (Asserachrome) according to manufacturer's instructions. Absorbance of each sample is determined spectrophotometrically, using Elx800 automated microplate reader (BIO TEK Instruments) and normalized to antigen standard curves. Mouse plasma samples were tested for the presence of anti-FIX Abs by ELISA, as described (Follenzi et al, 2004). Briefly, micro-titer plates are coated with recombinant human FIX (Pfizer) at 0.2 µg/well in 0.1 M carbonate buffer, pH 9.6. Three dilutions (1:500, 1:5000, 1:50,000) of mouse plasma are added and antibodies are detected with HRP-conjugated rabbit anti-mouse total Ig (Dako)., IgG (Sigma), IgG1, IgG2a, IgG2b (Serotech), IgE (Southern Biotech) Plates are reacted with H_2_O_2_ and ortho-phenylenediamine and read at 492 nm, using Elx800 automated microplate reader (BIO TEK Instruments). For quantification of IgG a standard curve of mouse IgG1 (BD) is tested in parallel. The limit of detection of the IgG Abs ELISA is 2 µg/ml. Mouse mast-cell protease-1 and histamine were determined on mouse plasma samples by ELISA (eBioscience or Immunotech, respectively), according to manufacturer instructions. To measure the titers of FIX-specific IgE, IgG were removed from plasma samples using protein G sepharose (GE Healthcare).

FIX activity was determined in an activated partial thromboplastin time (aPTT) assay employing Actin-FSL (Siemens) and human FIX-deficient plasma (Diagnostica Stago) and using a semi-automated coagulometer (BioMerieux). Plasma samples were tested at a 1:40 dilution and calibration curves were constructed with serial dilutions of pooled normal human plasma collected from 80 healthy volunteers and arbitrarily assigned a value of 100% FIX activity.

FIX inhibitor was determined by the Nijmegen modification of Bethesda assay (Verbruggen et al, 1995). The basic procedure is a 2-h incubation of the test sample with Imidazole-buffered (pH 7.4) normal human pooled plasma with FIX activity of 100%. A reference mixture of test-specific normal human pooled plasma with FIX-deficient plasma is also prepared. After incubation the residual FIX activity (the relative percentage of FIX activity in the test sample mixture compared to the reference mixture) is converted into BU/ml, where one BU is defined as the amount of inhibitor that yields 50% residual FIX activity. Inhibitor activity of test sample is read from a semi-logarithmic plot representing the correlation between residual FIX activity (logarithmic) and inhibitor activity (linear). The regression line is fully defined by 100% residual FIX activity with no inhibitor and 50% residual FIX activity with 1 BU/ml.

**The paper explained**

PROBLEM:

Neutralizing antibodies (inhibitors) against the therapeutic clotting factor prevents factor replacement therapy and increases morbidity and mortality in haemophilia. Immune tolerance induction (ITI) protocols, based on repeated administration of the clotting factor, can be attempted. However, especially for haemophilia B, the success of ITI in eradicating inhibitors is limited and there is a risk of anaphylaxis and nephrotic syndrome.

RESULTS:

We show that liver-directed gene therapy can reverse pre-existing anti-factor IX (FIX) humoral immunity in haemophilia B mice, eradicate FIX inhibitors, stably reconstitute FIX expression and restore haemostasis to near-normal levels. This outcome was due to: (i) contraction of FIX-specific plasma cell (PC) and memory B-cell pools and (ii) induction of FIX-specific regulatory T cells, which inhibit T-cell help required for generation of PCs and re-activation of memory B-cells. Inhibitors-positive haemophilia B mice showed anaphylactoid reactions after intravenous administrations of recombinant FIX protein, mimicking clinical observations. On the contrary, inhibitor eradication by gene therapy did not cause such adverse reactions.

IMPACT:

Our study suggests that hepatic gene transfer-based ITI may provide an effective alternative treatment to eradicate inhibitors in haemophilia B patients and stably treat the underlying disease. Furthermore, this strategy may be broadly applicable to reversal of antibodies in other conditions.

### B- and T-cell assays

Mice were euthanized at the expected time point, spleen, BM and LNs were collected and processed to single-cell suspension. *In vitro* assays were performed in RPMI 1640 (Lonza) medium, 10% fetal bovine serum with the addition of l-glutamine 2 mM, 2-mercaptoethanol 0.5 mM, non-essential amino acids, Sodium pyruvate 1 mM, penicilium 100 U/ml and streptomycin 100 µg/ml. Anti-FIX Abs secreting B cells were enumerated by elispot culturing LN, spleen and BM cells for 24 h in elispot plate (Multiscreen HA, Millipore) coated with 1 µg of FIX/well. Anti-FIX Abs specific spot-forming units were detected by HRP-conjugated anti-mouse IgG (Sigma) and visualized in red using AEC solution (0.05% AEC and 0.015% H_2_O_2_ in 0.05 M acetate buffer, pH 5.5). Spots were counted by Eli.Expert elispot Reader and analysed by Eli.Analyze software V5.1 (A.EL.VIS).

T cells proliferation was measured by ^3^H-thymidine incorporation. Briefly, B-cell-depleted splenocytes were negatively selected removing B cells by B220-µbeads (Miltenyi) magnetic sorting and cultured for 5 days in the presence of increasing doses of FIX. ^3^H-thymidine was added for the last 16 h of culture.

To detect activation of B_MEM_, PCs (CD138^+^) were depleted from LN cells by positive selection by anti-Pe µbeads (Miltenyi) of Pe-conjugated anti-CD138 (BD) labelled PC. PC-depleted LN cells were cultured for 5 days in the presence of FIX at concentrations ranging from 10 µg/ml to 10 pg/ml and transferred for an additional day of culture in a elipot plate coated with coated with 1 µg of FIX/well.

Immune-phenotyping was performed with the following monoclonal antibodies: APC-conjugated anti-CD4, Pe-conjugated CD25, FITC-conjugated anti-CD19, PerCp-conjugated anti-B220, PB-conjugated anti-IgD, Pe-Cy7-conjugated anti-IgM, FITC-conjugated anti-CD5, biotin-conjugated anti-CD1d, APC-conjugated streptavidin (all from BD Biosciences) or efluor-780-conjugated streptavidin (e Bioscience) and, biotin-conjugated recombinant human FIX (Pfizer). The conjugation reaction was performed using biotin-XX-NHS ester (Molecular Probe), according to manufacturer's instructions. Regulatory T and conventional T cells were FACS-sorted by MoFlo (Beckman Coulter).

Suppressive activity of Tregs and Tconv, isolated at the indicated time point from LV-FIX-treated inhibitor positive haemophilia B mice was evaluated co-culturing them with B-cell-depleted LN cells (at ratio 0.5:1) isolated from mice pre-immunized with FIX or OVA in IFA.

IL-10 secreting regulatory B cells were enumerated stimulating B-cell negatively purified from the spleen after T cells depletion by CD90.2 µbeads (Miltenyi). After 3 days of *in vitro* stimulation with 10 µg/ml LPS (Sigma) (polyclonal stimulation) or 10 µg/ml anti-mouse CD40 (clone 1C10; eBioscience) and plate-bound FIX (Ag-specific stimulation), B cells were cultured for 3 h in the presence of 10 ng/ml phorbol 12-myristate 13-acetate (PMA), 150 ng/ml ionomycin calcium salt and 10 µg/ml Brefeldin A. IL-10 producing B cells were quantified by intracellular staining using anti-mouse IL-10 Pe-conjugated (eBioscience). Immuno-stained cells were analysed with a FACSCanto flow cytometer equipped with Diva software (BD Biosciences).

### VCN determination

Vector DNA was quantified as follows: gDNA was extracted from liver samples by using Maxwell 16 Tissue DNA Purification Kit (Promega) according to manufacturer's instructions. VCN was quantified by qPCR using primers (HIV sense: 5′-TACTGACGCTCTCGCACC-3′; HIV antisense: 5′-TCTCGACGCAGGACTCG-3′) and a probe (FAM 5′-ATCTCTCTCCTTCTAGCCTC-3′) against the primer binding site region of LVs. Endogenous DNA amount was quantified by a primer/probe set against the murine β-actin gene (β-Act sense: 5′-AGAGGGAAATCGTGCGTGAC-3′; β-Act antisense: 5′-CAATAGTGATGACCTGGCCGT-3′; β-Act probe: VIC 5′-CACTGCCGCATCCTCTTCCTCCC-3′). Copies per genome were calculated as described above. The standard curve was generated by using samples with previously determined LV copies by Southern blot analysis.

### Statistical analysis

All statistical analyses were performed using the 2-tailed Student *t* test at *p* < 0.05 level of significance.

## Supplementary material

As a service to our authors and readers, this journal provides supporting information supplied by the authors. Such materials are peer reviewed and may be re-organized for online delivery, but are not copy-edited or typeset. Technical support issues arising from supporting information (other than missing files) should be addressed to the authors.

**Figure S1.** Anti-FIX IgG1, IgG2a, IgG2b and IgE measured by ELISA as indicated, in plasma samples collected from haemophilia B mice at 4 (**A**) and 10 (**B**) weeks after FIX immunization. The positivity cut-off (mean of 3 samples from a naïve haemophilia B mouse+3 standard deviations) is indicated. Data are mean ± SEM. FIX-specific IgG producing plasma cells (PCs) were enumerated by Elispot assay in the draining LNs, Spleen and Bone marrow isolated from haemophilia B mice at 4 (**C**) and 10 (**D**) weeks after FIX immunization.

**Figure S2.** Anti-FIX IgG Abs measured by ELISA (**A**,**B**,**E**,**F**) and FIX inhibitors measured by Bethesda Assay (**C**,**D**,**G**,**H**) in plasma samples collected from LV-FIX treated mice at the indicated times after FIX immunization. (**A**–**C**) mice non-responders to gene therapy (NR, detectable Bethesda units (BU)/ml 4 and/or FIX activity <1% of normal) after early treatment, as indicated. (**B**,**D**) responders mice are shown for comparison (whose mean is plotted in Fig 2A,B). (**E**,**G**) mice non-responders to gene therapy (NR, detectable BU/ml and/or FIX activity <1% of normal) after late treatment, as indicated. (**F**,**H**) responders mice are shown for comparison (whose mean is plotted in Fig 2E,F). Data from single mice are plotted.

**Figure S3.** Anti-FIX IgG1 (**A**,**D**), IgG2a (**B**,**E**), IgG2b (**C**,**F**), measured by ELISA in plasma samples collected from haemophilia B mice at the indicated times after FIX immunization. Mice were immunized and subsequently injected with saline (*n* = 4, red line) or 1 × 10^9^ TU/mouse of LV-FIX (*n* = 5 responders, green line) at 4 weeks after immunization (**A**,**B**,**C**), as indicated (early treatment). Mice were immunized and subsequently injected with saline (*n* = 3, red line) or 1 × 10^9^ TU/mouse of LV-FIX (*n* = 5 responders, green line) at 10 weeks after immunization (**D**,**E**,**F**), as indicated (late treatment). Data are presented as mean ± SEM.

**Figure S4.** FIX expression (black and green lines) is plotted as percentage of the maximal observed value throughout the experimental follow up (% max) after LV-FIX gene therapy in naïve haemophilia B mice (black line, mean 3 independent experiments, reported in Cantore et al, 2012), or after LV-FIX gene therapy in anti-FIX Abs positive haemophilia B mice (green line, mean of responder mice, as shown in Fig 2C,G), performed at 4 weeks (**A**; early treatment) or 10 weeks (**B**; late treatment) after FIX immunization. Anti-FIX IgG (red line) concentration is plotted as percentage of the value determined before performing gene therapy (% initial).

**Figure S5.** (**A**) FIX expression measured by ELISA on plasma samples collected from naïve haemophilia B mice treated with i.v. administration of recombinant FIX protein (200 IU/kg, black line, *n* = 4; 20 IU/kg, grey line, *n* = 4) at the indicated times after injection. Data are presented as mean ± SEM.

**Figure S6.** (**A**) Schematic diagram of the gating strategy used to identify interleukin-10 (IL-10) producing B cells (B10). B10 are comprised within the CD1d^hi^ and CD5^+^ subset among the B-cell population (CD19^+^B220^+^). Percentage of CD1d^hi^ and CD5^+^ B cells (**B**,**D**) and percentage of IL-10 producing B cells (by intracellular staining) within the CD1d^hi^ and CD5^+^ B cells (**C**,**E**), upon polyclonal stimulation with lipopolysaccharide (LPS) or FIX-specific stimulation (anti-CD40 and plate-bound FIX) as indicated. Mice were immunized and subsequently injected with saline (*n* = 2, white bars) or 1 × 10^9^ TU/mouse of LV-FIX (*n* = 3 responders, black bars) at 10 weeks after immunization. The frequency of B10 was evaluated at 12 weeks after gene therapy (22 weeks after immunization). Agematched naive haemophilia B mice were used as controls (*n* = 2, grey bars).

## References

[b1] Annoni A, Brown BD, Cantore A, Sergi LS, Naldini L, Roncarolo MG (2009). In vivo delivery of a microRNA-regulated transgene induces antigen-specific regulatory T cells and promotes immunologic tolerance. Blood.

[b2] Astermark J, Lacroix-Desmazes S, Reding M (2008). Inhibitor development. Haemophilia: The official journal of the World Federation of. Hemophilia.

[b3] Astermark J, Santagostino E, Keith Hoots W (2010). Clinical issues in inhibitors. Haemophilia.

[b4] Benson G, Auerswald Gn, Elezovic I, Lambert T, Ljung R, Morfini M, Remor E, Salek S (2012). Immune tolerance induction in patients with severe hemophilia with inhibitors: Expert panel views and recommendations for clinical practice. Eur J Haematol.

[b5] Berntorp E, Shapiro A (2012). Modern haemophilia care. Lancet.

[b6] Brown BD, Cantore A, Annoni A, Sergi LS, Lombardo A, Della Valle P, D'Angelo A, Naldini L (2007). A microRNA-regulated lentiviral vector mediates stable correction of hemophilia B mice. Blood.

[b7] Brown BD, Venneri MA, Zingale A, Sergi Sergi L, Naldini L (2006). Endogenous microRNA regulation suppresses transgene expression in hematopoietic lineages and enables stable gene transfer. Nat Med.

[b8] Cantore A, Nair N, Della Valle P, Di Matteo M, Matrai J, Sanvito F, Brombin C, Di Serio C, D'Angelo A, Chuah M (2012). Hyperfunctional coagulation factor IX improves the efficacy of gene therapy in hemophilic mice. Blood.

[b9] Cao O, Dobrzynski E, Wang L, Nayak S, Mingle B, Terhorst C, Herzog RW (2007). Induction and role of regulatory CD4 + CD25+ T cells in tolerance to the transgene product following hepatic in vivo gene transfer. Blood.

[b10] DiMichele D (2007). Inhibitor development in haemophilia B: An orphan disease in need of attention. Br J Haematol.

[b11] DiMichele D, Hoots W, Pipe S, Rivard G, Santagostino E (2007). International workshop on immune tolerance induction: Consensus recommendations. Haemophilia: Official J World Fed Hemophilia.

[b12] Ewenstein B, Takemoto C, Warrier I, Lusher J, Saidi P, Eisele J, Ettinger L, DiMichele D (1997). Nephrotic syndrome as a complication of immune tolerance in hemophilia B. Blood.

[b13] Fillatreau S, Sweenie C, McGeachy M, Gray D, Anderton S (2002). B cells regulate autoimmunity by provision of IL-10. Nat Immunol.

[b14] Finkelman F, Rothenberg M, Brandt E, Morris S, Strait R (2005). Molecular mechanisms of anaphylaxis: Lessons from studies with murine models. J Allergy Clin Immunol.

[b15] Finn J, Ozelo M, Sabatino D, Franck H, Merricks E, Crudele J, Zhou S, Kazazian H, Lillicrap D, Nichols T (2010). Eradication of neutralizing antibodies to factor VIII in canine hemophilia A after liver gene therapy. Blood.

[b16] Follenzi A, Battaglia M, Lombardo A, Annoni A, Roncarolo MG, Naldini L (2004). Targeting lentiviral vector expression to hepatocytes limits transgene-specific immune response and establishes long-term expression of human antihemophilic factor IX in mice. Blood.

[b17] Follenzi A, Naldini L (2002). HIV-based vectors. Preparation and use. Methods Mol Med.

[b18] Fox R, Neufeld E, Bennett C (2006). Rituximab for adolescents with haemophilia and high titre inhibitors. Haemophilia: Official J World Fed Hemophilia.

[b19] Hausl C, Ahmad R, Sasgary M, Doering C, Lollar P, Richter Gn, Schwarz H, Turecek P, Reipert B (2005). High-dose factor VIII inhibits factor VIII-specific memory B cells in hemophilia A with factor VIII inhibitors. Blood.

[b20] High KA (2012). The gene therapy journey for hemophilia: Are we there yet. Blood.

[b21] Iikuni N, Lourenco E, Hahn B, La Cava A (2009). Cutting edge: Regulatory T cells directly suppress B cells in systemic lupus erythematosus. J Immunol.

[b22] Li X-M, Srivastava K, Grishin A, Huang C-K, Schofield B, Burks W, Sampson H (2003). Persistent protective effect of heat-killed Escherichia coli producing ‘engineered,’ recombinant peanut proteins in a murine model of peanut allergy. J Allergy Clin Immunol.

[b23] Mannucci P, Tuddenham E (2001). The hemophilias–from royal genes to gene therapy. New Engl J Med.

[b24] Markusic DM, Hoffman BE, Perrin GQ, Nayak S, Wang X, LoDuca PA, High KA, Herzog RW (2013). Effective gene therapy for hemophilic mice with pathogenic factor IX antibodies. EMBO Mol Med.

[b25] Mathias M, Khair K, Hann I, Liesner R (2004). Rituximab in the treatment of alloimmune factor VIII and IX antibodies in two children with severe haemophilia. Br J Haematol.

[b26] Matrai J, Cantore A, Bartholomae CC, Annoni A, Wang W, Acosta-Sanchez A, Samara-Kuko E, De Waele L, Ma L, Genovese P (2011). Hepatocyte-targeted expression by integrase-defective lentiviral vectors induces antigen-specific tolerance in mice with low genotoxic risk. Hepatology.

[b27] Matzinger P (1994). Tolerance, danger, and the extended family. Ann Rev Immunol.

[b28] Mingozzi F, Liu YL, Dobrzynski E, Kaufhold A, Liu JH, Wang Y, Arruda VR, High KA, Herzog RW (2003). Induction of immune tolerance to coagulation factor IX antigen by in vivo hepatic gene transfer. J Clin Invest.

[b29] Nathwani A, Tuddenham E, Rangarajan S, Rosales C, McIntosh J, Linch D, Chowdary P, Riddell A, Pie A, Harrington C (2011). Adenovirus-associated virus vector-mediated gene transfer in hemophilia B. New Eng J Med.

[b30] Reipert BM, van Helden PM, Schwarz HP, Hausl C (2007). Mechanisms of action of immune tolerance induction against factor VIII in patients with congenital haemophilia A and factor VIII inhibitors. Br J Haematol.

[b31] Scott D, Lozier J (2012). Gene therapy for haemophilia: Prospects and challenges to prevent or reverse inhibitor formation. Br J Haematol.

[b32] Shlomchik M, Weisel F (2012). Germinal center selection and the development of memory B and plasma cells. Immunol Rev.

[b33] Sun B, Kulis M, Young S, Hobeika A, Li S, Bird A, Zhang H, Li Y, Clay T, Burks W (2010). Immunomodulatory gene therapy prevents antibody formation and lethal hypersensitivity reactions in murine pompe disease. Mol Ther: J Am Soc Gene Ther.

[b34] Verbruggen B, Novakova I, Wessels H, Boezeman J, van den Berg M, Mauser-Bunschoten E (1995). The Nijmegen modification of the Bethesda assay for factor VIII:C inhibitors: Improved specificity and reliability. Thromb Haemost.

[b35] Verma D, Moghimi B, LoDuca P, Singh H, Hoffman B, Herzog R, Daniell H (2010). Oral delivery of bioencapsulated coagulation factor IX prevents inhibitor formation and fatal anaphylaxis in hemophilia B mice. Proc Natl Acad Sci USA.

[b36] Wang L, Zoppe M, Hackeng TM, Griffin JH, Lee KF, Verma IM (1997). A factor IX-deficient mouse model for hemophilia B gene therapy. Proc Natl Acad Sci USA.

[b37] Warrier I, Bussel J, Valdez L, Barbosa J, Beardsley D (1997). Safety and efficacy of low-dose intravenous immune globulin (IVIG) treatment for infants and children with immune thrombocytopenic purpura. Low-Dose IVIG Study Group. J Pediatr Hematol/Oncol.

[b38] Yoshizaki A, Miyagaki T, DiLillo D, Matsushita T, Horikawa M, Kountikov E, Spolski R, Poe J, Leonard W, Tedder T (2012). Regulatory B cells control T-cell autoimmunity through IL-21-dependent cognate interactions. Nature.

