# Supplementary material for: Liver gene therapy by lentiviral vectors reverses anti-factor IX pre-existing immunity in haemophilic mice
Source: EMBO Mol Med. 2013 Sep 16;5(11):1684–97. doi: 10.1002/emmm.201302857 (PMC3840485; doi:10.1002/emmm.201302857)
Supplement: Supplementary file 1 [file emmm0005-1684-SD1.pdf]

EMBO Molecular Medicine

Liver gene therapy by lentiviral vectors reverses anti-factor IX pre-existing immunity in hemophilic mice

Andrea Annoni, Alessio Cantore, Patrizia Della Valle, Kevin Goudy, Mahzad Akbarpour, Fabio Russo, Sara Bartolaccini, Armando D'Angelo, Maria Grazia Roncarolo and Luigi Naldini

Corresponding author: Luigi Naldini, San Raffaele Telethon Institute for Gene Therapy

Review timeline:

Submission date:	08 April 2013
Editorial Decision:	13 May 2013
Revision received:	18 July 2013
Accepted:	08 August 2013

Transaction Report:

(Note: With the exception of the correction of typographical or spelling errors that could be a source of ambiguity, letters and reports are not edited. The original formatting of letters and referee reports may not be reflected in this compilation.)

Editor: Roberto Buccione

1st Editorial Decision 13 May 2013

Thank you for the submission of your manuscript to EMBO Molecular Medicine. We have now heard back from the three Reviewers whom we asked to evaluate your manuscript.
You will see that all three Reviewers are generally supportive of you work although a number of concerns have been raised that prevent us from considering publication at this time.

Reviewer 1 notes that OVA-injected, but not saline-injected animals, show an IgG decline over time and suggests that the number of controls animals should be increased to rule out non-specific effects. S/he also wonders whether the response to high dose FIX is a Type 1 hypersensitivity. Finally, Reviewer 1 mentions that to confirm the relevance of Tregs, adoptive transfer experiments should be performed. A few other issues are listed, which require your intervention.

Reviewer 2 is generally satisfied with the manuscript but notes that at variance with this manuscript, the back-to-back submission "Liver gene therapy by lentiviral vectors reverses anti-Factor IX pre-existing immunity in hemophilic mice" by Annoni et al. (EMM-2013-02857) reports elimination of neutralising antibodies with AAV-mediated transfer at only 6% of normal FIX levels. Reviewer 2 feels, and I agree, that this should be addressed and discussed appropriately.

Reviewer 3 requires clarification with respect to data reported in figure 2A-D. S/he also lists a number of issues that require your intervention including proper citation of previous work and elucidation of some unclear or imprecise statements.

Considered all the above, while publication of the paper cannot be considered at this stage, we would be pleased to consider revised submission, with the understanding that the Reviewers' concerns must be fully addressed, with additional experimental data where appropriate and that

acceptance of the manuscript will entail a second round of review.

Please note that it is EMBO Molecular Medicine policy to allow a single round of revision only and that, therefore, acceptance or rejection of the manuscript will depend on the completeness of your responses included in the next, final version of the manuscript.

As you know, EMBO Molecular Medicine has a "scooping protection" policy, whereby similar findings that are published by others during review or revision are not a criterion for rejection. However, I do ask you to get in touch with us after three months if you have not completed your revision, to update us on the status. Please also contact us as soon as possible if similar work is published elsewhere.

I look forward to seeing a revised form of your manuscript as soon as possible.

***** Reviewer's comments *****

Referee #1 (General Remarks):

The authors address the problem of inhibitor-positive hemophilia in a gene therapy mouse model. The authors conclude that LV mediated gene therapy has a tolerisation effect through induction of specific Treg cells. These data are largely observational however, and the immunological mechanism of Treg induction and memory B cell depletion is not elucidated.
1. OVA injected animals (Fig2A) also show a decline in IgG over time, although saline injected do not. The numbers of control animals should be increased to match that of the FIX group to rule out a non-specific effect (4 out of 16 FIX injected animals did not respond).
2. Copy number data should be shown and correlated with therapeutic effect. Furthermore, data confirming lack of FIX expression in non-hepatic tissues (including APCs) should be shown. The minimum dose required for efficacy should also be determined.
3. Can the authors confirm experimentally that the response to high dose FIX is truly a Type 1 hypersensitivity ? What happens if lower doses are used.....id this regimen produce comparable levels of FIX to that of gene therapy treated animals ?
4. labeling of Fig 4 axes are inconsistent between panels.
5. In fig 6 it is not clear how many mice the data is collected from, or whether the samples were pooled. The SEM looks remarkable small if these were triplicates from different experimental animals.
6. To confirm the importance of Tregs, the authors must perform adoptive transfer experiments. The long term duration of response in terms of Treg depletion should be determined. They should also determine whether there is any change in the number of IL-10 producing B cells.

Referee #2 (General Remarks):

This study shows that lentiviral vectors can be used express FIX in the liver of hemophilic mice and to eliminate neutralizing antiFIX antibody responses in immunized FIX-deficient mice. This effect was mediated by depletion of FIX-specific plasma cells and B memory cells, at least in part due to induction of Tregs by the transgene product. It is interesting to note that while the majority of treated mice showed reversal of inhibitory antibodies, about 25% did not respond and this was associated with a lower vector copy number. This suggests that the ability to abrogate neutralizing antibodies is dependent on the absolute level of transgene-induced FIX expression. Measured FIX levels in responders was approximately 100% that of normal, suggesting this is the required level for the response. However, in the accompanying paper by Markusic et al, elimination of neutralizing antibodies with AAV-mediated transfer occurred at only 6% of normal FIX levels. If both papers are published, the authors should address this potential discrepancy.For instance, is it possible that the ability of lentiviral vectors to induce the Treg response is less than that seen with AAV (at a given FIX level) ? Is one vector system intrinsically more potent that the other in terms of eliminating inhibitors ? This will certainly be a question the readers will be interested in. If this cannot be

addressed experimentally, at least the authors should acknowledge this difference in the studies and discuss the possible ramifications and interpretations. Otherwise, this is a nice study with well performed experiments and a medically important result.

Referee #3 (General Remarks):

Annoni et al convincingly show that they can attenuate established anti-factor IX antibodies with liver-directed gene transfer using lentivirus vectors in hemophilia B mice. This is an important problem in humans for various reasons, particularly that factor IX inhibitors prevent proper therapy for bleeding and/or prophylaxis against bleeding, and inhibitors to factor IX can be associated with anaphylaxis and/or nephrotic syndrome in the event of repeat exposure to factor IX. In fact, the anaphylaxis in humans was first noted in patients undergoing standard desensitization with human factor IX proteins. Thus, a safe, effective approach to treating these inhibitors would be welcome.

Some comments and questions that arise in my review of this manuscript follow.

1. C57BL/ mice are well known not to be highly responsive to human factor IX protein as an immunogen, at least delivered intravenously as is the route of administration for factor IX in hemophilia B patients.
2. It is a bit of an overstatement to call inhibitors to factor IX "life-threatening" as in the Abstract.
3. Lower dose immune tolerance regimens have been described and might be mentioned in the Introduction.
4. Introduction may mention early references by Warrier I, et al and Ewenstein B, et al that describe anaphylaxis and nephrotic syndrome in factor IX inhibitor patients undergoing ITI.
5. In results would mention on page 4 that the hemophilia B mice are in the C57Bl/6 background; this is sufficiently important to readers who follow this field that it should be stated here, however briefly.
6. On page 8, last sentence of 2nd paragraph deems anti-factor IX antibodies of low titer detected by ELISA to be non-inhibitory because the Bethesda inhibitor assay is negative. The discrepancy between the ELISA result and the Bethesda inhibitor assay result is a function of the fact that ELISA tests detect ALL antibodies that bind a fixed antigen target, while an assay based on neutralization of clotting function does not register a result until >50% of the factor IX is neutralized. This is intrinsic to the way the tests are done, and it should not be stated that the affinity is necessarily low or the antibodies do not neutralize factor IX. It is probably better to state that the residual titers of factor IX antibodies detected by ELISA method were not high enough to reach the threshold required to inhibit clotting enough to register on the Bethesda assay. I would not speculate on the affinity of the antibody without more data (which is not really necessary for publication).
7. In the Results, with reference to Figure 2A-D, why is there detectable FIX antigen and activity at the 6 week time point with significant anti-FIX antibody detectable by ELISA and Bethesda assay? Also, why is there 25% factor IX concomitant with 800 microgram/ml IgG antibody and ~30 BIAU neutralizing antibody titer in late treatement group (2E-H) but in the early treatment group (2A-D) there is 10% factor IX concomitant with 100 microgram/ml IgG antibody and ~3 BIAU neutralizing antibody titer in early treatment group (2A-D)?

Minor/stylistic concerns:

1. F8 or F9 to designate the genes for factor VIII or factor IX respectively should be italicized.
2. Factor VIII and factor IX are not proper nouns and need not be capitalized.
3. Comcomitantly, plasma factor IX levels and clotting activity rose (not raised) to 50-100% of normal.
4. Last line of Results on page 4: presume Authors refer to IgG1 isotype, not "G1"?
5. Second DiMichelle reference: strike "Haemophilia : the offical journal of the World Federation of Hemophilia" (not required)

1st Revision - authors' response 18 July 2013

We want to thank the reviewers for their overall positive appraisal of our manuscript and their helpful and constructive suggestions. In order to address them, we have performed additional experiments and modified the manuscript accordingly. Below please find a *point-by-point response* to the reviewers' comments.

Referee #1 (General Remarks):

The authors address the problem of inhibitor-positive hemophilia in a gene therapy mouse model. The authors conclude that LV mediated gene therapy has a tolerisation effect through induction of specific Treg cells. These data are largely observational however, and the immunological mechanism of Treg induction and memory B cell depletion is not elucidated.

1. OVA injected animals (Fig2A) also show a decline in IgG over time, although saline injected do not. The numbers of control animals should be increased to match that of the FIX group to rule out a non-specific effect (4 out of 16 FIX injected animals did not respond).

We performed statistical analysis and included it in Figure 2A. Although the average concentration of anti-FIX IgG in LV-OVA treated mice appeared lower in the last time point of analysis as compared to all earlier measurements, it still did not significantly differ from that found in saline-injected mice at the same time point. Moreover, inhibitor titers remained superimposable between the two groups for the entire analysis including the last time point (see Figure 2B). We agree with the Reviewer that the number of LV-OVA control animals is low; indeed the observed decrease in average concentration was mainly due to the last data point from one mouse (#9.10), while the other two LV-OVA control mice did not show a decrease, as it can be seen in the graph below plotting the data for individual mice. Some similar upward and downward fluctuations were also observed sporadically in the larger group of saline injected mice. Thus, there is little reason to suspect a non-specific effect of the LV-OVA treatment, which in any case should have manifested several weeks before in the earlier time points (as observed for the LV-FIX). If the Reviewer thinks that we should include the graph showing individual mice in a Supporting Information Figure, we can do it.

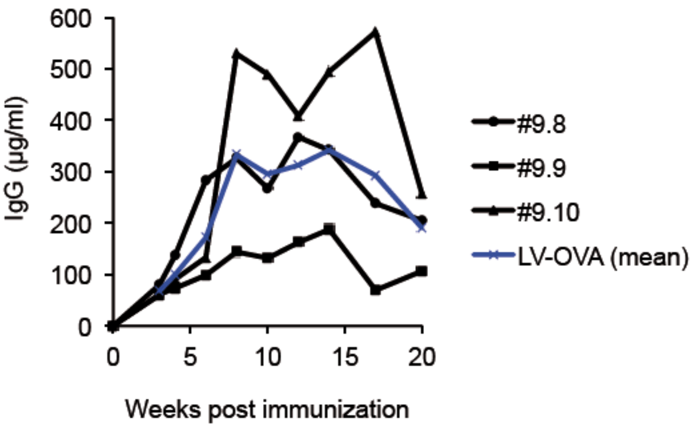

2. *Copy number data should be shown and correlated with therapeutic effect.*

Vector copy number data are shown in Table 1 and in Supporting Information Figure 2. We have observed a lower vector copy number in those mice in which gene therapy was not effective in reversing the anti-FIX humoral response (Supporting Information Figure 2). We mention the data in the results section ("Intriguingly, NR mice had significantly lower vector content in the liver than responder mice at the end of the experiments") and discuss a possible correlation with the observed effect in the discussion ("It is possible that NR mice did not achieve the threshold of FIX expression required to halt the immune response, due to variability in transduction efficiency. This hypothesis is supported by the finding of an average lower vector content in the liver of NR vs. responder mice").

Furthermore, data confirming lack of FIX expression in non-hepatic tissues (including APCs) should be shown.

We have previously shown that lentiviral vectors containing the hepatocyte-specific ET promoter and microRNA 142 target sequences stringently target transgene expression to hepatocytes and abolish any residual transgene expression directly from the transduced vector in antigen presenting cells of the liver and spleen, using a reporter GFP transgene (Brown et al., Nat Med 2006, Brown et al., Blood 2007). These data and the papers reporting them are referenced in the Introduction.

The minimum dose required for efficacy should also be determined.

We and others have reported that even a low level of hepatocyte-targeted FIX expression (1% of normal) and a low percentage of transduced hepatocytes (2%) (Matrai et al., Hepatology 2011; Martino et al., Plos One 2009) are sufficient to induce Tregs and tolerance to the delivered transgene in naïve mice. Since in the current work we aimed to reverse a pre-existing humoral immune response, we postulated that high antigen doses would be required to control the B-cell response. This assumption was based on previous reports that exposure to antigens in the μg/ml range of concentration was unable to re-stimulate memory B cells *in vitro* (Hausl et al., Blood 2005). We thus treated inhibitors-positive hemophilia B mice with LV doses (about 10^9 TU/ml) able to provide >50% of normal FIX levels in the circulation (corresponding to 2.5 μg/ml). Using these LV doses we obtained a 70-75% positive response, in terms of reversal of inhibitor titers and recovery of FIX activity. Because the non-responder mice had significantly lower average vector content in the liver than the responder mice at the end of the experiments (see Supporting Information Figure 2, as mentioned above), we interpret the failure to respond as due to the failure to reach a threshold of FIX expression required to halt the immune response, due to variability in transduction efficiency. For this reason, we did not perform further experiments at lower LV doses, as it is unlikely that lower doses would be effective, unless transient depletion of antibodies or plasma cells is performed (see below). Because further dose-response studies would require large cohorts of mice and long periods of observation, we would consider that such studies better belong to the follow-up of the current work, as stated in the discussion: "Further studies exploring the dose-dependent response of gene therapy for inhibitor reversal will address this point".

Relevant to this point, please consider also our response to a question raised by Reviewer 2. Both papers by Markusic et al. and ours show that FIX-specific memory B cells are less prone to re-activate upon exposure to FIX concentration higher than 300 ng/ml (corresponding to 6% of normal levels). An apparent discrepancy between our manuscript and the companion manuscript by Markusic is that, while in the latter it is reported that reversal of FIX inhibitors occurs with AAV-mediated gene therapy at only 6% of normal FIX levels, we report that the same outcome is achieved at 50-100% of normal FIX levels upon LV-mediated gene therapy. However, it should be noted that there are several potentially important model-dependent differences between the two studies, due to the different genetic background of the FIX knock out mice used and the different types of immunization protocols adopted (intravenous injections of the antigen in Markusic vs. subcutaneous antigen administration in the presence of incomplete Freund's adjuvant in our study). Likely for these reasons, anti-FIX IgG concentrations are lower in the Markusic study, reaching

approximately 30 μg/ml at the time of gene therapy administration, while in our work they reach approximately 200 μg/ml (see Figure 2A and 2E). As we postulate that reversal of the B-cell response is achieved only above a certain threshold of circulating FIX concentration, it is possible that the initial bioavailability of FIX is lower in our case, due to the higher concentration of binding anti-FIX antibodies. Thus, pre-treatment regimens aiming to transiently deplete circulating antibodies and/or B cells before gene therapy may also increase the success rate and, possibly, decrease the required vector dose, by favoring early antigen bioavailability. In order to clarify this point we added the above text in the discussion of the revised manuscript.

3. Can the authors confirm experimentally that the response to high dose FIX is truly a Type 1 hypersensitivity?

As we could not detect anti-FIX IgE in immunized mice, we referred to the adverse events observed in mice receiving FIX protein injections as anaphylactoid reactions, implying that these reactions were not strictly classifiable as type-I hypersensitivity. In order to improve our ability to detect anti-FIX IgE in the ELISA, we repeated the assays after removal of IgG from plasma samples using protein G sepharose, thus avoiding competition for the plate-bound FIX antigen, as we mention in the materials and methods section of the revised manuscript. Even after IgG depletion, the plasma of immunized mice resulted negative to anti-FIX IgE, indicating that our immunization protocol in this strain of mice does not induce a detectable IgE response. Moreover, we did not find anti-FIX IgE in the plasma of mice even after ITI-like treatment by repeated FIX protein administrations. Thus, we cannot define the response to FIX protein as a type-I hypersensitivity, as it appears not to involve IgE. However, the signs were typical of anaphylaxis, acute, severe and even lethal in some cases. In order to better characterize experimentally the mechanism of response, we thus decided to measure plasma histamine levels in the mice treated with high-dose FIX protein injections. We now show in the revised Figure 3B that this response is mediated, at least in part, by release of histamine and mast-cell protease-1, thus suggesting that it represents an IgE-independent anaphylaxis. This type of anaphylaxis is IgG-dependent and involves both mast-cells and macrophages through engagement of their Fcγ receptors (Finkelman et al, J Allergy Clin Immunol 2005).

What happens if lower doses are used.....id this regimen produce comparable levels of FIX to that of gene therapy treated animals ?

In order to clarify this point, we have included in the revised manuscript a new experiment in which pre-immunized hemophilia B mice were treated by repeated intravenous administrations of recombinant FIX at 20 IU/kg. The results of this experiment indicate that even doses 10-fold lower than those previously tested (200 IU/kg) induce hypothermia and signs of anaphylactoid reactions, although to a lesser extent than observed at high doses. The results of this experiment are now shown in the revised Figure 3A. We added the following text in the results section of the revised manuscript: "We evaluated whether these adverse events also occur following a lower dose ITI-like regimen. We treated hemophilia B mice by repeated i.v. administrations of recombinant FIX at 20 IU/kg, starting 10 weeks after immunization (n=5). Even a 10-fold lower FIX dose induced hypothermia and signs of anaphylactoid reactions, although to a lesser extent than observed with higher doses (Figure 3A)".

We have also measured the levels of circulating FIX achieved after intravenous administration of recombinant FIX at 20 or 200 IU/kg in naïve hemophilia B mice at 1, 6, 24 and 48 hours after administration. The results of this experiment are now shown in Supporting Information Figure 5 in the revised manuscript. The following text has been added in the results section of the revised manuscript: "In order to determine the levels of circulating FIX achieved after a high- and low-dose of FIX protein administered for ITI-like regimens, we intravenously administered 200 or 20 IU/kg of recombinant FIX in naïve hemophilia B mice (n=4 *per* dose) and detected 46% and 2% of normal

FIX levels, respectively, 1 hour after administration. These levels declined by half in the next 6 hours and became almost undetectable after 2 days (Supporting Information Figure 5). As expected, in inhibitors-positive hemophilia B mice we could not detect circulating FIX, when assessed at 1 hour after protein administration".

4. labelling of Fig 4 axes are inconsistent between panels.

We adjusted Y axis scaling to allow direct comparison between early and late treatment data.

5. In fig 6 it is not clear how many mice the data is collected from, or whether the samples were pooled. The SEM looks remarkable small if these were triplicates from different experimental animals.

We performed a new suppression assay, after sorting conventional and regulatory T cells from a pool of two LV-FIX treated mice in a separate experiment. We now show the mean and range of the stimulation index from the two independent experiments, each one performed in triplicate, in the revised Figure 6C,D. The results confirm the higher suppressive capacity of induced regulatory T cells isolated from gene therapy treated mice in controlling the proliferative response to FIX as compared to that driven from an unrelated antigen.

6. To confirm the importance of Tregs, the authors must perform adoptive transfer experiments. The long term duration of response in terms of Treg depletion should be determined.

We have previously shown that transgene-specific Tregs induced upon systemic delivery of a microRNA 142-regulated LV can transfer tolerance to naïve recipients. In particular, the tolerance transfer capacity was confined to the CD4+CD25+ T-cell subset (Annoni et al., Blood 2009). To investigate a role of Tregs in the control of a pre-existing immune response, we performed Tregs depletion after LV-FIX gene therapy in inhibitors-positive hemophilia B mice. When performed early after gene therapy, Tregs depletion caused an increase in anti-FIX IgG and FIX inhibitors titer in those mice in which the humoral response was not yet shut down. Although these data support an involvement of Tregs in the reversal of the humoral response, we need to address the confounding factor that some mice do not respond *per se* to gene therapy. Thus, we either substantially expand the sample size or prolong the window of Tregs depletion in these challenging experiments to obtain statistically robust data or, preferably, we repeat them once we have established conditions leading to 100% response to gene therapy. As we are currently undertaking such endeavors we would prefer leaving these Treg depletion experiments to a follow-up study.

They should also determine whether there is any change in the number of IL-10 producing B cells.

Il-10 producing B cells (B10) have been shown to regulate inflammation and autoimmune diseases as well as innate and antigen-specific adaptive immune responses in some conditions (Fillatreau et al, Nat Immunol 2002; Yoshizaki et al, Nature 2012). We evaluated whether B10 are expanded in inhibitors-positive hemophilia B mice after gene therapy. We found no difference in the percentage of B10 in mice after LV-FIX treatment (n=3) as compared to saline-injected controls (n=2) and naïve hemophilia B mice (n=2), both upon a polyclonal (lipopolysaccharide, LPS) or FIX-specific stimulation (anti-CD40 and plate-bound FIX). These new data are shown in the Supporting

Information Figure 6. They suggest that B10 are unlikely to be responsible for the regulation of the FIX humoral response observed after gene therapy. We added the above text in the results section of the revised manuscript.

Referee #2 (General Remarks):

This study shows that lentiviral vectors can be used express FIX in the liver of hemophilic mice and to eliminate neutralizing antiFIX antibody responses in immunized FIX-deficient mice. This effect was mediated by depletion of FIX-specific plasma cells and B memory cells, at least in part due to induction of Tregs by the transgene product. It is interesting to note that while the majority of treated mice showed reversal of inhibitory antibodies, about 25% did not respond and this was associated with a lower vector copy number. This suggests that the ability to abrogate neutralizing antibodies is dependent on the absolute level of transgene-induced FIX expression. Measured FIX levels in responders was approximately 100% that of normal, suggesting this is the required level for the response. However, in the accompanying paper by Markusic et al, elimination of neutralizing antibodies with AAV-mediated transfer occurred at only 6% of normal FIX levels. If both papers are published, the authors should address this potential discrepancy. For instance, is it possible that the ability of lentiviral vectors to induce the Treg response is less than that seen with AAV (at a given FIX level) ? Is one vector system intrinsically more potent that the other in terms of eliminating inhibitors ? This will certainly be a question the readers will be interested in. If this cannot be addressed experimentally, at least the authors should acknowledge this difference in the studies and discuss the possible ramifications and interpretations. Otherwise, this is a nice study with well performed experiments and a medically important result.

The Reviewer raises an interesting point. We and others have reported that even a low level of hepatocyte-targeted FIX expression (1% of normal) and a low percentage of transduced hepatocytes (2%) (Matrai et al., Hepatology 2011; Martino et al., Plos One 2009) are sufficient to induce Tregs and tolerance to the delivered transgene in naïve mice. Since in the current work we aimed to reverse a pre-existing humoral immune response, we postulated that high antigen doses would be required to control the B-cell response. This assumption was based on previous reports that exposure to antigens in the µg/ml range of concentration was unable to re-stimulate memory B cells *in vitro* (Hausl et al., Blood 2005). We thus treated inhibitors-positive hemophilia B mice with LV doses (about 10^9 TU/ml) able to provide >50% of normal FIX levels in the circulation (corresponding to 2.5 µg/ml). Using these LV doses we obtained a 70-75% positive response, in terms of reversal of inhibitor titers and recovery of FIX activity. Because the non-responder mice had significantly lower average vector content in the liver than the responder mice at the end of the experiments (see Supporting Information Figure 2, as mentioned above), we interpret the failure to respond as due to the failure to reach a threshold of FIX expression required to halt the immune response, due to variability in transduction efficiency. For this reason, we did not perform further experiments at lower LV doses, as it is unlikely that lower doses would be effective, unless transient depletion of antibodies or plasma cells is performed.

Both papers by Markusic et al. and ours show that FIX-specific memory B cells are less prone to re-activate upon exposure to FIX concentration higher than 300 ng/ml (corresponding to 6% of normal levels). As the Reviewer points out, an apparent discrepancy between our manuscript and the companion manuscript by Markusic is that, while in the latter it is reported that reversal of FIX inhibitors occurs with AAV-mediated gene therapy at only 6% of normal FIX levels, we report that the same outcome is achieved at 50-100% of normal FIX levels upon LV-mediated gene therapy. However, it should be noted that there are several potentially important model-dependent differences between the two studies, due to the different genetic background of the FIX knock-out mice used and the different types of immunization protocols adopted (intravenous injections of the antigen in Markusic vs. subcutaneous antigen administration in the presence of incomplete Freund's adjuvant in our study). Likely for these reasons, anti-FIX IgG concentrations are lower in the Markusic study, reaching approximately 30 µg/ml at the time of gene therapy administration, while in our work they reach approximately 200 µg/ml (see Figure 2A and 2E). As we postulate that

reversal of the B-cell response is achieved only above a certain threshold of circulating FIX concentration, it is possible that the initial bioavailability of FIX is lower in our case, due to the higher concentration of binding anti-FIX antibodies. Thus, pre-treatment regimens aiming to transiently deplete circulating antibodies and/or B cells before gene therapy may also increase the success rate and, possibly, decrease the required vector dose, by favoring early antigen bioavailability.

In order to address and clarify this point we added the above text in the discussion of the revised manuscript.

Referee #3 (General Remarks):

Annoni et al convincingly show that they can attenuate established anti-factor IX antibodies with liver-directed gene transfer using lentivirus vectors in hemophilia B mice. This is an important problem in humans for various reasons, particularly that factor IX inhibitors prevent proper therapy for bleeding and/or prophylaxis against bleeding, and inhibitors to factor IX can be associated with anaphylaxis and/or nephrotic syndrome in the event of repeat exposure to factor IX. In fact, the anaphylaxis in humans was first noted in patients undergoing standard desensitization with human factor IX proteins. Thus, a safe, effective approach to treating these inhibitors would be welcome.

Some comments and questions that arise in my review of this manuscript follow.

1. C57BL/ mice are well known not to be highly responsive to human factor IX protein as an immunogen, at least delivered intravenously as is the route of administration for factor IX in hemophilia B patients.

We agree with the reviewer that hemophilia B mice in the C57Bl/6 genetic background are not highly responsive to FIX protein as immunogen. Indeed they mount a robust anti-FIX humoral immune response only upon subcutaneous administration of high doses of FIX in the presence of incomplete Freund's adjuvant, as described in the manuscript.

2. It is a bit of an overstatement to call inhibitors to factor IX "life-threatening" as in the Abstract.

We have removed "life-threatening" from the abstract and throughout the text.

3. Lower dose immune tolerance regimens have been described and might be mentioned in the Introduction.

We have now mentioned low-dose ITI regimens in the introduction of the revised manuscript. Moreover, we have included in the revised manuscript a new experiment in which pre-immunized hemophilia B mice were treated by repeated intravenous administrations of recombinant FIX at 20 IU/kg. The results of this experiment indicate that even doses 10-fold lower than those previously tested (200 IU/kg) induce hypothermia and signs of anaphylactoid reactions, although to a lesser extent than observed at high doses. The results of this experiment are now shown in the revised Figure 3A. We added the following text in the results section of the revised manuscript: "We

evaluated whether these adverse events also occur following a lower dose ITI-like regimen. We treated hemophilia B mice by repeated i.v. administrations of recombinant FIX at 20 IU/kg, starting 10 weeks after immunization (n=5). Even a 10-fold lower FIX dose induced hypothermia and signs of anaphylactoid reactions, although to a lesser extent than observed with higher doses (Figure 3A)".

4. Introduction may mention early references by Warrier I, et al and Ewenstein B, et al that describe anaphylaxis and nephrotic syndrome in factor IX inhibitor patients undergoing ITI.

We thank the Reviewer for the suggestion and have now included Warrier I, et al and Ewenstein B, et al references in the introduction of the revised manuscript.

5. In results would mention on page 4 that the hemophilia B mice are in the C57Bl/6 background; this is sufficiently important to readers who follow this field that it should be stated here, however briefly.

We added this information to the results section of the revised manuscript.

6. On page 8, last sentence of 2nd paragraph deems anti-factor IX antibodies of low titer detected by ELISA to be non-inhibitory because the Bethesda inhibitor assay is negative. The discrepancy between the ELISA result and the Bethesda inhibitor assay result is a function of the fact that ELISA tests detect ALL antibodies that bind a fixed antigen target, while an assay based on neutralization of clotting function does not register a result until >50% of the factor IX is neutralized. This is intrinsic to the way the tests are done, and it should not be stated that the affinity is necessarily low or the antibodies do not neutralize factor IX. It is probably better to state that the residual titers of factor IX antibodies detected by ELISA method were not high enough to reach the threshold required to inhibit clotting enough to register on the Bethesda assay. I would not speculate on the affinity of the antibody without more data (which is not really necessary for publication).

We thank the Reviewer for this insight. We have thus revised the presentation and discussion of this data as suggested by the Reviewer in the following sentence of the results section of the revised manuscript: "It is possible that the residual titers of binding anti-FIX Abs detected by immunocapture were not high enough to reach the threshold required to inhibit clotting enough to register on the Bethesda assay."

7. In the Results, with reference to Figure 2A-D, why is there detectable FIX antigen and activity at the 6 week time point with significant anti-FIX antibody detectable by ELISA and Bethesda assay? Also, why is there 25% factor IX concomitant with 800 microgram/ml IgG antibody and ~30 BIAU neutralizing antibody titer in late treatment group (2E-H) but in the early treatment group (2A-D) there is 10% factor IX concomitant with 100 microgram/ml IgG antibody and ~3 BIAU neutralizing antibody titer in early treatment group (2A-D)?

Figure 2 shows the data as mean and standard error of the mean of several mice. Indeed, when scrutinizing data mouse *per* mouse, we could correlate detectable FIX in the plasma with lower titers of anti-FIX binding antibodies. Data on anti-FIX IgG concentration and inhibitor titers are reported for single mice in Supporting Information Figure 2. For better clarity, we have added the following sentence to the Legend to Figure 2: "Data are presented as mean±SEM. ns: not significant; **: $p<0.01$; ***: $p<0.001$ (t test). When data were scrutinized for each individual mouse,

detectable FIX in the plasma correlated with lower level of anti-FIX binding antibodies (see also Supporting Information Figure 2).”

Minor/stylistic concerns:

1. F8 or F9 to designate the genes for factor VIII or factor IX respectively should be italicized.

The reviewer correctly underlines the proper way to refer to genes or proteins. Indeed in the introduction we refer to the proteins encoded by the genes (factor VIII, abbreviated FVIII and factor IX, abbreviated FIX). In the results section, first paragraph, we italicize *F9*, as we refer to the gene.

2. Factor VIII and factor IX are not proper nouns and need not be capitalized.

We have changed Factor VIII and Factor IX into factor VIII and factor IX throughout the text in the revised manuscript.

3. Comcomitantly, plasma factor IX levels and clotting activity rose (not raised) to 50-100% of normal.

We thank the reviewer for careful reading. We have changed accordingly throughout the text in the revised manuscript.

4. Last line of Results on page 4: presume Authors refer to IgG1 isotype, not "G1"?

Correct. We have changed “G1” with “IgG1” in the revised manuscript.

5. Second DiMichelle reference: strike "Haemophilia : the official journal of the World Federation of Hemophilia" (not required)

Journal names are automatically edited by software tools for managing references, as per the EMBO Molecular Medicine style. All the references for the journal “Haemophilia” include the sentence “the official journal of the World Federation of Hemophilia”.

2nd Editorial Decision 08 August 2013

Thank you for the submission of your revised manuscript to EMBO Molecular Medicine.

As mentioned in an earlier letter, we have experienced a delay due to the fact that Reviewer 1, whom we asked to re-evaluate your revised manuscript together with Reviewer 3 , is on vacation. We therefore asked Reviewer 3 also to check whether you had adequately replied to this Reviewer's

criticisms. As you will see the s/he is satisfied and is globally supportive.

We are thus pleased to inform you that your manuscript is accepted for publication and is being sent to our publisher to be included in the next available issue of EMBO Molecular Medicine.

Congratulations on your interesting work,

***** Reviewer's comments *****

Reviewer #3 (General Remarks):

The manuscript by Annoni et al on induction of tolerance to factor IX in mice with pre-existing antibodies (inhibitors), using lentiviral vectors, is revised to reflect critical remarks by myself and two other reviewers. I am satisfied that the revised manuscript addresses these criticisms in a satisfactory manner.

Reviewer #3 (Additional comments):

To the best of my ability to comprehend, both sets of Authors have replied to Reviewer 1's criticisms in a fully satisfactory way that adds value to each paper.
