# Supplementary material for: Liver gene therapy by lentiviral vectors reverses anti-factor IX pre-existing immunity in haemophilic mice
Source: EMBO Mol Med. 2013 Sep 16;5(11):1684–97. doi: 10.1002/emmm.201302857 (PMC3840485; doi:10.1002/emmm.201302857)
Supplement: Supplementary file 2 [file emmm0005-1684-SD2.pdf]

Liver gene therapy by lentiviral vectors reverses anti-factor IX pre-existing immunity in hemophilic mice

Andrea Annoni[1,#], Alessio Cantore[1,2,#], Patrizia Della Valle[3], Kevin Goudy[1], Mahzad Akbarpour[1,2], Fabio Russo[1], Sara Bartolaccini[1], Armando D'Angelo[3], Maria Grazia Roncarolo[1,2,*] and Luigi Naldini[1,2,*]

[1]San Raffaele Telethon Institute for Gene Therapy, San Raffaele Scientific Institute, Milan, Italy;
[2]Vita Salute San Raffaele University, Milan, Italy;
[3]Coagulation Service and Thrombosis Research Unit, San Raffaele Scientific Institute, Milan, Italy
# These authors contributed equally.
* These authors share senior authorship.

Supporting information:

- **Figure 1-6**

CORRESPONDING AUTHORS:
Luigi Naldini, San Raffaele Telethon Institute for Gene Therapy and ''Vita Salute San Raffaele'' University, Via Olgettina 58, 20132 Milano, Italy. E-mail: naldini.luigi@hsr.it; Phone: +39 02 2643 4681; Fax: +39 02 2643 4621.
Maria Grazia Roncarolo, San Raffaele Telethon Institute for Gene Therapy and ''Vita Salute San Raffaele'' University, Via Olgettina 58, 20132 Milano, Italy. E-mail: roncarolo.mariagrazia@hsr.it; Phone: +39 02 2643 4870; Fax: +39 02 2643 4668

Supporting Information Figure 1

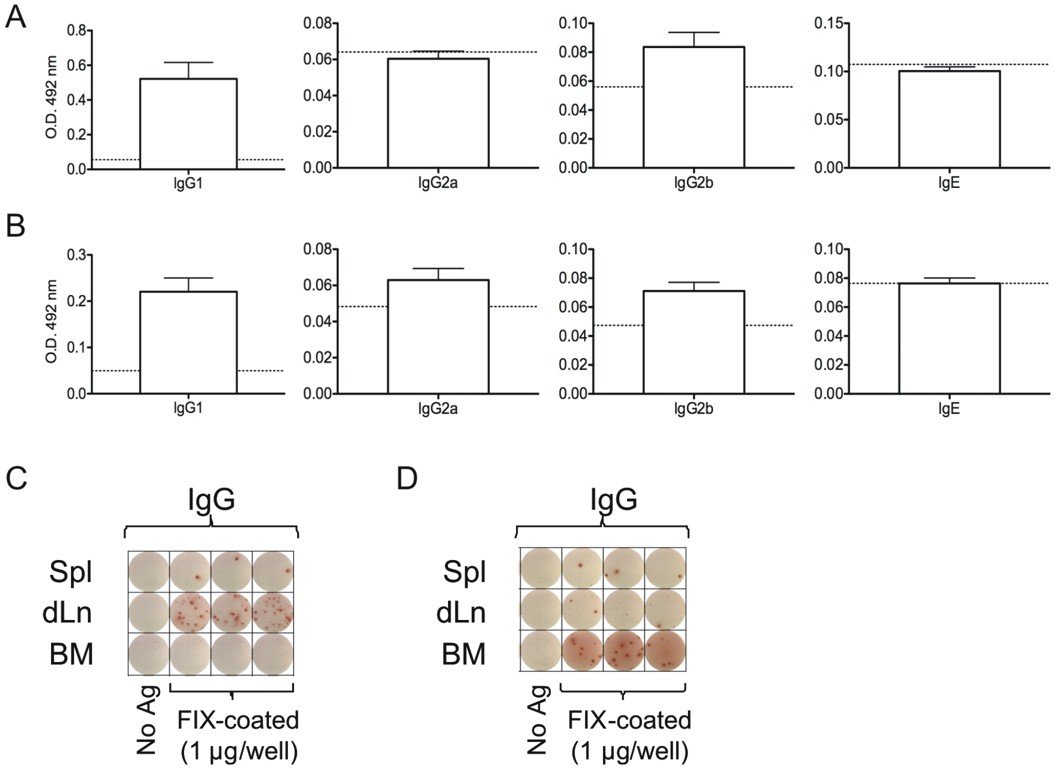

Supporting Information Figure 1. Anti-FIX IgG1, IgG2a, IgG2b and IgE measured by ELISA as indicated, in plasma samples collected from hemophilia B mice at 4 (**A**) and 10 (**B**) weeks after FIX immunization. The positivity cut-off (mean of 3 samples from a naïve hemophilia B mouse+3 standard deviations) is indicated. Data are mean±SEM. FIX-specific IgG producing plasma cells (PCs) were enumerated by Elispot assay in the draining LNs, Spleen and Bone marrow isolated from hemophilia B mice at 4 (**C**) and 10 (**D**) weeks after FIX immunization.

Supporting Information Figure 2

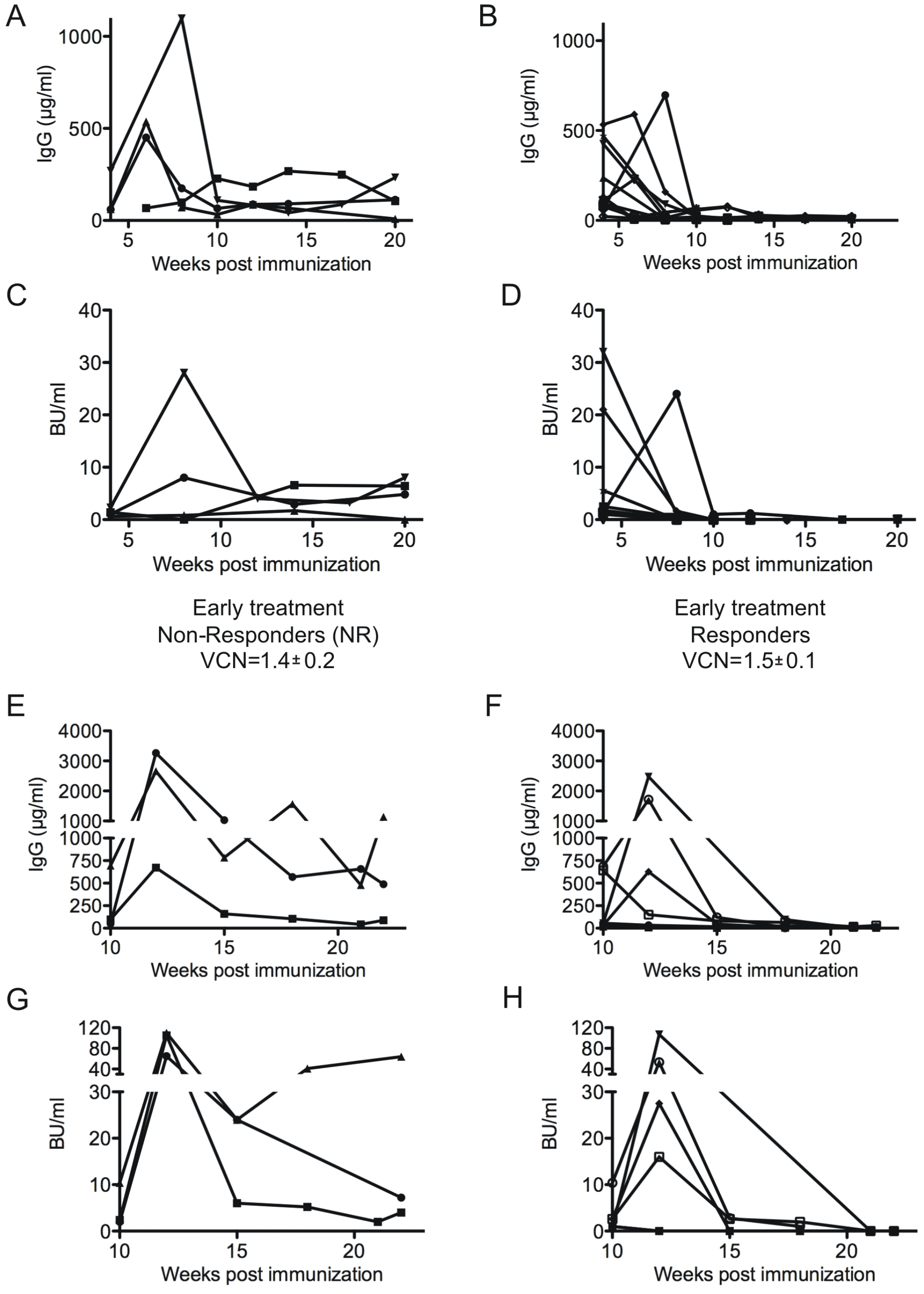

Supporting Information Figure 2. Anti-FIX IgG Abs measured by ELISA (**A,B,E,F**) and FIX inhibitors measured by Bethesda Assay (**C,D,G,H**) in plasma samples collected from LV-FIX treated mice at the indicated times after FIX immunization. (**A-C**) mice non responders to gene therapy (NR, detectable BU/ml

and/or FIX activity <1% of normal) after early treatment, as indicated. (**B,D**) responders mice are shown for comparison (whose mean is plotted in Figure 2A,B). (**E,G**) mice non responders to gene therapy (NR, detectable BU/ml and/or FIX activity <1% of normal) after late treatment, as indicated. (**F,H**) responders mice are shown for comparison (whose mean is plotted in Figure 2E,F). Data from single mice are plotted.

Supporting Information Figure 3

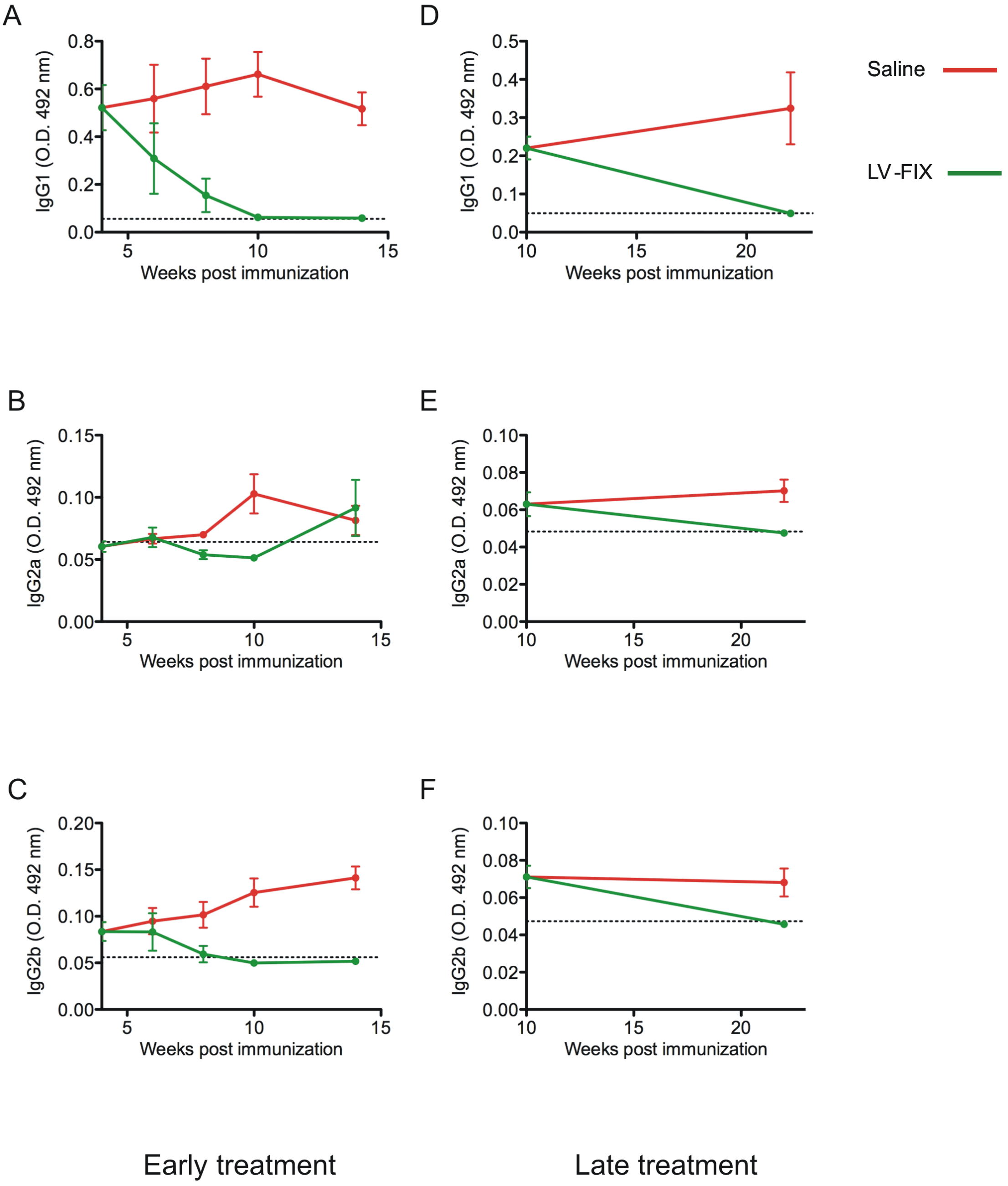

Supporting Information Figure 3. Anti-FIX IgG1 (**A,D**), IgG2a (**B,E**), IgG2b (**C,F**), measured by ELISA in plasma samples collected from hemophilia B mice at the indicated times after FIX immunization. Mice were immunized and subsequently injected with saline (n=4, red line) or 1x10^9 TU/mouse of LV-FIX (n=5 responders, green line) at 4 weeks after immunization (**A,B,C**), as indicated (early treatment). Mice were immunized and subsequently injected with saline (n=3, red line) or 1x10^9 TU/mouse of LV-FIX (n=5 responders, green line) at 10 weeks after immunization (**D,E,F**), as indicated (late treatment). Data are presented as mean±SEM.

Supporting information Figure 4

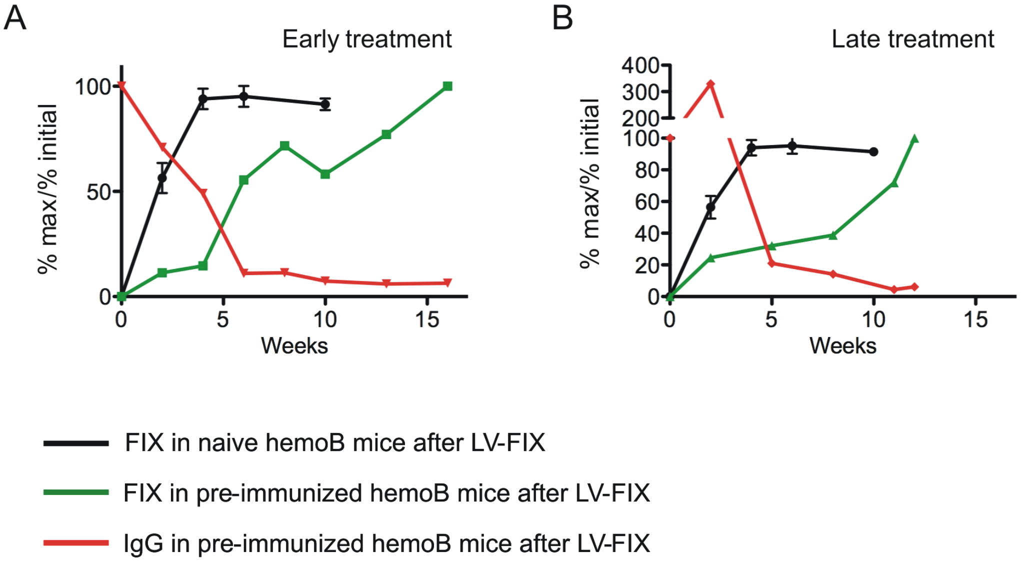

Supporting Information Figure 4. FIX expression (black and green lines) is plotted as percentage of the maximal observed value throughout the experimental follow up (% max) after LV-FIX gene therapy in naïve hemophilia B mice (black line, mean 3 independent experiments, reported in Cantore et al, 2012), or after LV-FIX gene therapy in anti-FIX Abs positive hemophilia B mice (green line, mean of responder mice, as shown in Figure 2C,G), performed at 4 weeks (**A**; early treatment) or 10 weeks (**B**; late treatment) after FIX immunization. Anti-FIX IgG (red line) concentration is plotted as percentage of the value determined before performing gene therapy (% initial).

Supporting Information Figure 5

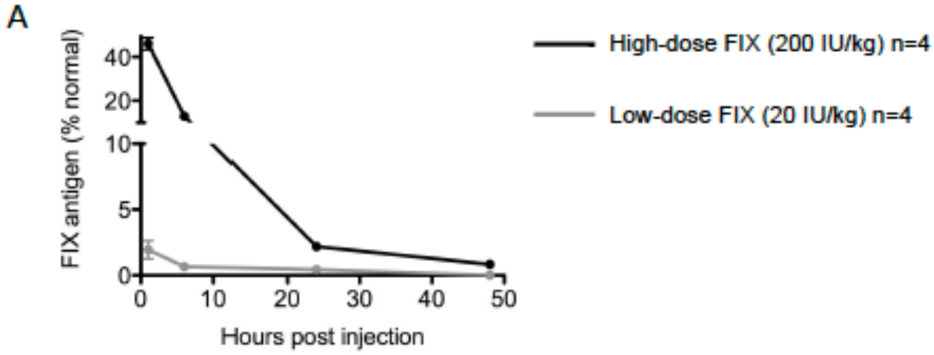

Supporting Information Figure 5. (**A**) FIX expression measured by ELISA on plasma samples collected from naïve hemophilia B mice treated with i.v. administration of recombinant FIX protein (200 IU/kg, black line, n=4; 20 IU/kg, grey line, n=4) at the indicated times after injection. Data are presented as mean±SEM.

Supporting information Figure 6

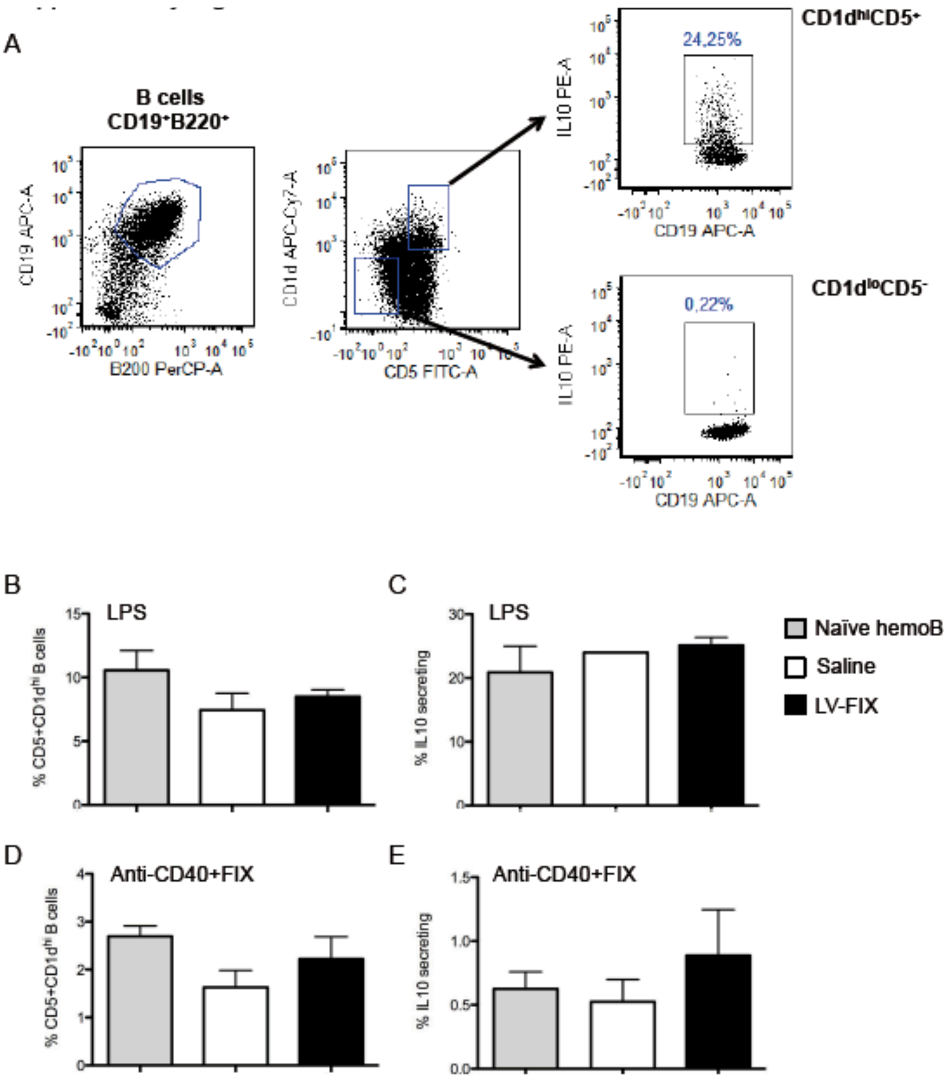

Supporting Information Figure 6. (**A**) Schematic diagram of the gating strategy used to identify interleukin-10 (IL-10) producing B cells (B10). B10 are comprised within the CD1d^{hi} and CD5^{+} subset among the B-cell population (CD19^{+}B220^{+}). Percentage of CD1d^{hi} and CD5^{+} B cells (**B,D**) and percentage of IL-10 producing B cells (by intracellular staining) within the CD1d^{hi} and CD5^{+} B cells (**C,E**), upon polyclonal stimulation with lipopolysaccharide (LPS) or FIX-specific stimulation (anti-CD40 and plate-bound FIX) as indicated. Mice were immunized and subsequently injected with saline (n=2, white bars) or 1x10^{9} TU/mouse of LV-FIX (n=3 responders, black bars) at 10 weeks after immunization. The frequency of B10 was evaluated at 12 weeks after gene therapy (22 weeks after immunization). Age-matched naïve hemophilia B mice were used as controls (n=2, grey bars).
